# Molecular Recognition of Diaryl Ureas in Their Targeted Proteins—A Data Mining and Quantum Chemical Study

**DOI:** 10.3390/molecules30051007

**Published:** 2025-02-21

**Authors:** Majed S. Aljohani, Xiche Hu

**Affiliations:** 1 Department of Chemistry and Biochemistry, University of Toledo, Toledo, OH 43606, USA; mssjohani@taibahu.edu.sa; 2 Department of Chemistry, College of Science Yanbu, Taibah University, Yanbu 30799, Saudi Arabia

**Keywords:** diaryl ureas, molecular recognition, protein kinases inhibitors, aromatic rings, rational drug design, hydrogen bonding, π–π stacking interactions, XH-π interaction, quantum chemical analysis

## Abstract

Diaryl ureas (DU) are a cornerstone scaffold in organic and medicinal chemistry, celebrated for their unique structural attributes and broad range of biomedical applications. Their therapeutic reach has broadened beyond kinase inhibition in cancer therapy to encompass diverse mechanisms, including modulation of chromatin remodeling complexes, interference with developmental signaling pathways, and inhibition of stress-activated protein kinases in inflammatory disorders. A critical element in the rational design and optimization of DU-based therapeutics is a detailed understanding of their molecular recognition by target proteins. In this study, we employed a multi-tiered computational approach to investigate the molecular determinants of DU–protein interactions. A large-scale data mining of the Protein Data Bank resulted in an in-house dataset of 158 non-redundant, high-resolution crystal structures of DU–protein complexes. This dataset serves as the basis for a systematic analysis of nonbonded interactions, including hydrogen bonding, salt bridges, π–π stacking, CH-π, cation–π, and XH-π interactions (X = OH, NH, SH). Advanced electronic structure calculations at the B2PLYP/def2-QZVP level are applied to quantify the energetic contributions of these interactions and their roles in molecular recognition of diaryl ureas in their target proteins. The study led to the following findings: central to the molecular recognition of diaryl ureas in proteins are nonbonded π interactions—predominantly CH-π and π–π stacking—that synergize with hydrogen bonding to achieve high binding affinity and specificity. Aromatic R groups in diaryl ureas play a pivotal role by broadening the interaction footprint within hydrophobic protein pockets, enabling energetically favorable and diverse binding modes. Comparative analyses highlight that diaryl ureas with aromatic R groups possess a more extensive and robust interaction profile than those with non-aromatic counterparts, emphasizing the critical importance of nonbonded π interactions in molecular recognition. These findings enhance our understanding of molecular recognition of diaryl ureas in proteins and provide valuable insights for the rational design of diaryl ureas as potent and selective inhibitors of protein kinases and other therapeutically significant proteins.

## 1. Introduction

Urea and its derivatives are celebrated scaffolds in organic and medicinal chemistry, offering unparalleled structural flexibility and pharmacological utility [1,2]. The urea molecule, comprising a carbonyl group flanked by two amine groups, functions as both a hydrogen bond donor and acceptor. This unique duality enables urea to form precise, directional interactions with biological targets. The hydrogen-bonding capability of the urea group also significantly enhances the solubility and permeability of urea-containing drug molecules. Furthermore, the urea molecule has been derivatized with various functional groups to improve its binding affinity to target proteins [3]. These derivatives, commonly known as ureas, are widely used in drug design due to their ability to increase potency, enhance selectivity, reduce toxicity, and fine-tune the physicochemical properties of pharmacophores [1,3,4,5,6].

**Figure 1:**
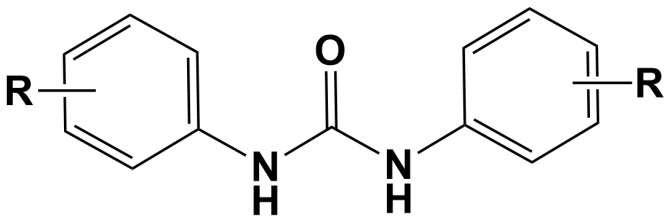
Chemical structure of diaryl ureas (DU), the R groups can be aromatic or non-aromatic.

Among the numerous urea derivatives, diaryl ureas (DU) have emerged as a dominant subclass [3,4], particularly for their applications in oncology [5,6]. The general structure of diaryl ureas is depicted in Figure 1. Structurally, diaryl ureas are defined by the incorporation of two aromatic systems flanking the urea core. Different linkage and functional groups can be added to these aromatic rings to improve both the binding capacity and spatial reach inside the binding pockets of targeted proteins.

As shown in Figure 1, the urea moiety of DU plays a crucial role in forming anchoring hydrogen bonds, while the aromatic rings enhance binding by driving key nonbonded π interactions within hydrophobic pockets of target proteins, solidifying diaryl ureas as a highly versatile scaffold in medicinal chemistry. The seminal discovery of sorafenib, a diaryl urea-based type II tyrosine kinase inhibitor, marked a transformative moment in oncology, providing proof-of-concept for diaryl ureas as selective inhibitors of VEGFR, PDGFR, and related targets [5]. The success of subsequent derivatives, including regorafenib and linifanib, further demonstrated the versatility of diaryl ureas in targeting the ATP-binding pockets of kinases in DFG-out conformations. Beyond their anticancer properties, diaryl ureas exhibit broad-spectrum bioactivity, including antimicrobial, antiviral, and anti-inflammatory effects, establishing them as multitarget agents with high translational potential [1,4,6,7,8,9,10].

Recent advancements in structural biology, particularly high-resolution X-ray crystallography, have resulted in the structural determination of hundreds of DU-bound protein complexes. This lays the structural foundation for our investigation of molecular recognition of diaryl ureas in their target proteins here. It is nonbonded interactions that mediate molecular recognition between diaryl ureas and their target proteins, as in all ligand–protein complexes [11,12]. Traditionally, the consideration of nonbonded interactions mainly included hydrogen bonding and salt bridge interactions. However, in recent years, more and more evidence suggests that π-moiety involved interactions, such as π–π stacking interactions [13], CH-π interactions [14], cation–π interactions [15], are just as important as hydrogen bonding and salt bridges [16,17,18]. As is performed in Ref. [19], hereinafter, all these π-moiety involved interactions will be collectively termed “nonbonded π-interactions”.

Nonbonded interactions are essentially a juxtaposition of several elements, including electrostatic interactions, exchange–repulsion interactions, induction, and dispersion forces. Of these, dispersion forces are the primary source of attraction between neutral molecules. These forces originate from correlated fluctuations in electron density between interacting monomers, known as intermolecular correlation effects, with correlation energy often being comparable in magnitude to the overall interaction energy. As a result, the inclusion of electron correlation is essential in any accurate electronic structure calculation of nonbonded complexes. Wavefunction-based methods, such as second-order Møller–Plesset perturbation theory (MP2) and coupled cluster with single, double, and perturbative triple excitations [CCSD(T)], have been widely employed to capture these effects [17]. MP2 provides a balance between computational cost and accuracy, recovering a significant portion of the correlation energy [20,21]. In contrast, CCSD(T), often considered the “gold standard” of quantum chemistry, delivers highly accurate interaction energies by providing a more complete treatment of electron correlation. However, its computational cost scales steeply, making it impractical for large biomolecular systems. Configuration interaction (CI) methods, although capable of including electron correlation, are generally unsuitable for treating nonbonded interactions due to the lack of size consistency in truncated CI approaches and the prohibitively high computational cost of full CI calculations.

Historically, density functional theory (DFT) was limited in its ability to describe nonbonded interactions due to the lack of dispersion forces in standard exchange-correlation functionals. However, the development of dispersion-corrected DFT (DFT-D) methods in the mid-2000s has significantly improved its accuracy in describing nonbonded interactions [22]. These methods introduce empirical dispersion corrections, typically of the form C6/R6, where C6 coefficients are parameterized based on atomic pairs. Over successive iterations, methods such as DFT-D2 and DFT-D3 have improved accuracy and reduced empiricism by refining the determination of dispersion coefficients. In this context, the double hybrid density functional B2PLYP [23,24], combined with an atom-pairwise dispersion correction using the Becke–Johnson damping scheme (D3BJ) [22], has been applied here to quantify the strengths of nonbonded interactions. This choice is based on a systematic benchmarking of dispersion-corrected DFT methods against the highly accurate CCSD(T) method. It identified the RIJK RI-B2PLYP-D3/def2-QZVP implementation as one of the most accurate and computationally efficient DFT methods for treating nonbonded interactions [17].

In this study, we employed a multi-tiered computational approach to investigate the molecular determinants of DU–protein interactions. A large-scale data mining of the Protein Data Bank resulted in an in-house dataset of 158 non-redundant, high-resolution crystal structures of DU–protein complexes. This dataset serves as the basis for a systematic analysis of nonbonded interactions, including hydrogen bonding, salt bridges, π–π stacking, CH-π, cation–π, and XH-π interactions (X = OH, NH, SH). Advanced electronic structure calculations at the B2PLYP-D3/def2-QZVP level are applied to quantify the energetic contributions of these interactions and their roles in molecular recognition of diaryl ureas in their target proteins.

Through data mining, structural analysis, and quantum chemical calculations, this work aims to develop a mechanistic understanding of DU–protein interactions. Insights gained will inform the rational design of diaryl ureas as potent inhibitors targeting key proteins implicated in disease pathways. Particular emphasis is placed on the role of aromatic R groups, which enhance binding by expanding the interaction footprint and facilitating diverse nonbonded π interactions. The findings underscore the therapeutic potential of diaryl ureas for developing targeted therapies in cancer and other protein-mediated diseases.

The remainder of this article is structured as follows. The Results and Discussion section presents the findings of this study. Section 2.1 outlines the data mining process employed to identify and curate a dataset of 158 non-redundant DU–protein complexes, establishing a robust foundation for subsequent analyses. Section 2.2 examines the binding modes of these complexes, focusing on the prevalence and contributions of key nonbonded interactions, including CH-π interactions, hydrogen bonding, π–π stacking, and cation–π interactions. Section 2.3 introduces a curated library of 102 representative 3D binding motifs, which highlights the structural diversity and versatility of diaryl ureas in engaging residues inside protein binding pockets. Section 2.4 provides a comparative analysis of molecular determinants for DU binding between diaryl ureas with aromatic and non-aromatic R groups, exploring the distribution of energetic contributions from various nonbonded interactions to diaryl ureas binding across entire protein complexes. The Theory and Methods section details the procedures for data mining of DU-binding proteins from the Protein Data Bank and provides specific information on the B2PLYP/def2-QZVP electronic structure calculations used to quantify nonbonded interactions in DU–protein complexes. Finally, the Conclusion offers a concise summary of the study’s key findings.

## 2. Results and Discussion

### 2.1. Data Mining and Structural Analysis

The data mining of the PDB databank resulted in the discovery of 150 ligands with diaryl urea moiety. These ligands are associated with 158 non-redundant complexes. Table A1 provides a detailed summary of 158 non-redundant DU–protein complexes, each identified by a unique ligand ID (Column 1) and a corresponding PDB ID (Column 3). It lists the protein targets (Column 2) and the X-ray crystallographic resolution (Column 4), along with the reported K_i_, K_d_, and IC_50_ values when available. The proteins listed in Table A1 consist of 61 unique proteins that span a diverse range of functional types, including mainly protein kinases, enzymes, and receptors commonly involved in signal transduction and cellular regulation. Many of these proteins, such as mitogen-activated protein kinases (MAPKs), cyclin-dependent kinases (CDKs), and receptor tyrosine kinases, are frequently targeted in cancer therapy due to their roles in cell cycle regulation and apoptosis. Diaryl ureas behave as type II kinase inhibitors in DFG-out kinase conformation. The diaryl urea moiety was found to occupy a hydrophobic pocket adjacent to the ATP binding site [25]. Other notable proteins include carbonic anhydrase, glycogen phosphorylase, and dihydroorotate dehydrogenase, which are enzymes involved in metabolism and cellular respiration.

The chemical composition of 150 diaryl ureas was systematically analyzed. It was found that the R groups of 54 ligands do not contain any aromatic rings (corresponding to 56 complexes, 35.4%), whereas the R groups of 96 ligands contain at least one aromatic ring (102 complexes, 64.6%). For easy reference, they will be designated as non-aromatic R groups and aromatic R groups, respectively. Given the aromatic nature of diaryl ureas overall, we hypothesized that nonbonded π interactions (i.e., π–π stacking interactions, cation–π interactions, cation–π interactions, and XH-π interactions (X = O, N and S) may play an important role in molecular recognition of diaryl ureas binding proteins. Naturally, both the diaryl rings and aromatic rings in the R groups may have a significant contribution to the overall binding of diaryl ureas due to the formation of multiple nonbonded π interactions with the targets’ residues. These hypotheses will be tested by analyzing the modes of nonbonded interactions between diaryl ureas and their surrounding protein residues below.

### 2.2. Structural and Binding Mode Analysis

We have carried out a comprehensive analysis of the binding modes of diaryl ureas, focusing on the role of nonbonded interactions in protein–ligand recognition. The analysis explores how different R groups influence binding modes and specificity through three layers of investigation: a global binding mode analysis to capture overall trends, a focused examination of diaryl ureas with non-aromatic R groups, and a comparative analysis of diaryl ureas with aromatic R groups. The objective of this comparative analysis is to study the effect of R groups on the molecular recognition of diaryl ureas in proteins.

#### 2.2.1. Comprehensive Binding Mode Analysis of All DU–Protein Complexes

In this comprehensive analysis, we assess the binding modes of diaryl ureas in all available structures. Table 1 provides a comprehensive summary of nonbonded interactions observed in DU–protein complexes. The table lists the average occurrences of each interaction type, highlighting hydrogen bonding, CH-π interactions, π–π stacking, and NH-π interactions as key contributors to binding affinity.

As expected, hydrogen bonding emerges as a fundamental interaction for the diaryl ureas, occurring in nearly all complexes with an average of 4.0 bonds per complex. Specifically, the urea moiety contributes to the hydrogen binding of 128 complexes (81.0% of total complexes). The carbonyl oxygen of the urea moiety functions as a hydrogen bond acceptor, while the nitrogen atoms act as donors, establishing strong and directional interactions critical for molecular recognition. On average, the urea moiety forms 2.1 hydrogen bonds with surrounding residues, with the oxygen of the urea moiety as the most frequent participant. It accepts at least one hydrogen bond in 111 complexes (70.3%). In most cases, the urea moiety’s oxygen accepts hydrogen bonds from the protein’s backbone nitrogen atoms, particularly from charged residues such as aspartic acid and lysine.

Urea moiety also acts as a frequent hydrogen bond donor. Based on our alignment method, it was found that one nitrogen donates at least one hydrogen bond in 95 cases (60.1%), while the other nitrogen donates at least one hydrogen bond in 103 complexes (65.2%). Overall, two nitrogen atoms form hydrogen bonds in 88 complexes (55.7%) altogether. Three primary hydrogen bonding patterns were identified across the complexes analyzed:

(1) Dual Hydrogen Bonds: This pattern involves the nitrogen atoms of the urea moiety simultaneously donating hydrogen bonds to oxygen atoms of nearby residues. This pattern exists in 79 complexes (50.0% of total complexes) with a distribution: i. main chain (3, 3.8%), ii. side chain (76, 96.2%). In the case of side chain interaction, the most frequent residue is glutamic acid (E), which forms dual hydrogen bonding in 68 cases. In addition, the dual hydrogen-bonding mode was formed by aspartic acid in 10 cases.
(2) Ni-Oi Motif: In this motif, both the nitrogen and oxygen atoms of the urea moiety engage with a single residue, forming a dual interaction site. This configuration was less common (occurring in two complexes) but contributed to binding stability where observed.
(3) Ni+2-Oi Motif: This pattern involves a nitrogen atom two residues away from the original interacting residue, leading to a secondary layer of hydrogen bonding. It adds further stability in a limited number (12 complexes) of cases.

This simultaneous hydrogen bond donating and accepting capacity of the urea moiety contributes significantly to the binding specificity and strength of diaryl urea inhibitors.

The importance of aromatic groups is evidently revealed. CH-π interactions are the most frequent nonbonded π interactions, averaging 15.4 instances per complex. These interactions arise when the hydrophobic side chains of non-polar amino acids, such as alanine and valine, align closely with the aromatic rings of the diaryl urea moiety, stabilizing the complex through van der Waals forces. π–π stacking interactions occur at an average of 3.0 instances per complex. These interactions involve face-to-face or edge-to-face alignments between aromatic rings from both the diaryl urea and protein residues, such as phenylalanine, contributing additional binding stability. Cation–π interactions, while less frequent with an average of 1.2 per complex, involve positively charged residues like lysine and arginine interacting with the electron-rich π-cloud of the aromatic rings. Despite their lower occurrence, they still play a role in binding energy contribution.

**Figure 2:**
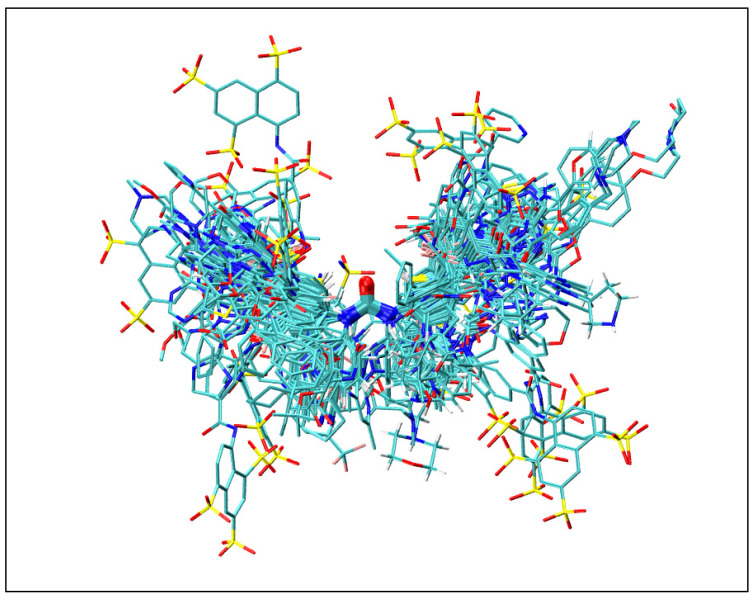
Alignment of all diaryl urea molecules extracted from the X-ray crystal structures of 158 diaryl ureas binding proteins listed in Table A1, with the urea moiety as the reference. Color coding: C (cyan), N (blue), O (red), and S (yellow). This figure is generated by the program VMD 1.9.3 [26].

Together, these nonbonded interactions—hydrogen bonding, along with CH-π, π–π stacking, and cation–π—work in concert to enhance the binding affinity of diaryl ureas, making them promising candidates for targeted protein inhibition in drug design. As shown in Figure 2, the 3D structures of all 158 diaryl ureas binding proteins (Table A1) were aligned by superimposition of the urea moiety using the VMD program [26]. The introduction of R groups containing multiple aromatic and non-aromatic groups with variable link length to diaryl urea moiety could give ligands added spatial flexibility to reach many more residues in the binding pocket of targets. Furthermore, the figure illustrates the wide spatial extent spanned by the diaryl urea derivatives. The spatial reach and orientation of aromatic groups in the diaryl urea derivatives were dependent upon the type and length of links. The latter can be easily adjusted so that the derivatized diaryl ureas can reach different regions in the binding pocket of the protein targets. This derivatization can modify the binding affinity and selectivity by gaining new nonbonded π interactions, including π–π stacking interactions, CH-π interaction, and cation–π interaction.

#### 2.2.2. Diaryl Ureas with Non-Aromatic R Groups

In this section, we analyze nonbonded interactions involving diaryl ureas with non-aromatic R groups. The latter are typically simple alkyl or hydrogen substituents that lack aromatic rings. As a result, the interactions formed by these compounds with protein targets are limited to primarily non-polar interactions and weaker bonding interactions. The analysis revealed that diaryl ureas with non-aromatic R groups bind to proteins with an average of 21.5 nonbonded interactions. The diaryl urea moiety is responsible for an average of 18.3 interactions. About 91.3% (16.7 interactions) of these interactions are nonbonded π interactions. In contrast, the non-aromatic R groups are responsible only for an average of 3.2 interactions. Table 2 provides a detailed summary of the nonbonded interactions in diaryl ureas with non-aromatic R groups, highlighting the types and frequencies of nonbonded interactions observed in all 56 complexes.

The binding mode analysis reveals that CH-π interactions dominate the interaction profile, being present in 100% of the complexes. As shown in Table 2, the average number of CH-π interactions is 9.3 (+0.8) per complex, highlighting their central role in the binding of these compounds. These interactions predominantly occur between the hydrophobic side chains of non-polar amino acids, such as alanine (A), valine (V), leucine (L), and isoleucine (I), and the aromatic rings of the diaryl urea moiety.

In terms of hydrogen bonding, the urea moiety forms 1.6 hydrogen bonds on average, observed in 63.2% of complexes (see Table 2). These bonds are typically formed between the oxygen of the urea moiety, which acts as a hydrogen bond acceptor, and its interacting residues. Additionally, the nitrogen atoms of the urea moiety serve as hydrogen bond donors. These hydrogen bonds stabilize the complex but are less extensive than those seen with aromatic R groups.

NH-π interactions were also significant, present in all complexes with an average of 3.2 interactions. Other important interactions include π–π stacking interactions in 91.1% of complexes (average of 2.3 interactions) and cation–π interactions in 46.4% (0.5 interactions). Less frequent interactions such as OH-π interactions (71.4%, 1.1 interactions) and SH-π interactions (25%, 0.3 interactions) were also observed. Notably, salt bridge interactions are observed in 23.2% of complexes with an average count of 0.3.

The non-aromatic R groups themselves make a small contribution to the overall binding, as reflected in the data from Table 2. These groups are responsible for an average of 2.1 hydrogen bonds, a small contribution to cation–π interactions (averaging 0.5 interactions per complex), and 0.8 CH-π interactions.

#### 2.2.3. Diaryl Ureas with Aromatic R Groups

In this section, nonbonded interactions involving diaryl ureas with aromatic R group(s) are analyzed and compared with the non-aromatic R group above. As mentioned earlier, 96 diaryl ureas contain at least one aromatic moiety in one or two of the R groups. These 96 ligands are associated with 102 protein–ligand complexes available from PDB. The binding mode analysis of this class resulted in the finding that diaryl ureas with aromatic R groups interact with targeted proteins extensively, with an average of 35.9 nonbonded interactions. On average, 20.4 interactions are formed by the diaryl urea moiety, and 17.9 (87.7%) of these interactions are nonbonded π interactions. Table 3 presents a detailed summary of the nonbonded interactions in diaryl ureas with aromatic R groups.

As shown in Table 3, CH-π interactions remain the most frequent interaction type, appearing in 100% of the complexes, with an average of 10.8 and 7.9 interactions per complex coming from the diaryl urea moiety and the aromatic R groups, respectively. The aromatic R groups significantly increase the binding strength due to the additional aromatic rings, which allow for greater engagement with hydrophobic residues in the binding pocket. The CH-π interactions in these complexes are thus more extensive and contribute more strongly to the overall binding compared to those observed in the non-aromatic R group complexes. It is worth noting that multiple CH-π interactions are introduced by aromatic R groups with residues that do not interact with diaryl urea moiety. This is observed in all 100 complexes (100%). Moreover, aromatic R groups can also engage in CH-π interactions with residues that also interact with diaryl urea moiety.

Hydrogen bonding is also more prevalent in diaryl ureas with aromatic R groups. As seen in Table 3, the urea moiety forms 2.5 hydrogen bonds on average, occurring in 87.3% of complexes. Moreover, the aromatic R groups themselves contribute 1.6 hydrogen bonds, further stabilizing the complex.

In addition to CH-π interactions, the aromatic R groups introduce significant additional π–π stacking interactions, which are present in 74.5% of complexes, with an average of 1.4 π–π interactions per complex (see Table 3). These interactions occur between the aromatic rings in the R groups and aromatic residues in the protein binding pocket, such as phenylalanine (F) and tryptophan (W). The presence of these aromatic rings facilitates additional binding contacts and strengthens the protein–ligand interaction.

The contribution of the aromatic R groups to cation–π interactions is also notable. These interactions occur in 38.2% of complexes and account for an average of 0.6 interactions per complex. This increase in cation–π interactions is due to the additional aromatic rings, which enable interactions with the positively charged side chains of lysine (K) and arginine (R), leading to further stabilization of the complex. Additionally, the diaryl urea moiety is involved in cation–π interactions in 77.5% of cases (1.0 interaction per complex). In comparison, cation–π interactions in non-aromatic R groups were present only in 46.4% of complexes, with a lower average of 0.5 interactions per complex (see Table 2).

Furthermore, NH-π interactions are frequently observed in diaryl ureas with aromatic R groups, with 2.8 interactions per complex in 98% of complexes (see Table 3). These interactions arise from the nitrogen atoms in the urea moiety interacting with the aromatic residues in the protein target.

Overall, aromatic R groups dramatically enhance the binding strength and specificity of diaryl ureas, as shown in Table 3, by introducing a broader range of energetically favorable interactions. These include enhanced CH-π, cation–π, and π–π stacking interactions, as well as increased hydrogen bonding. In contrast, non-aromatic R groups rely primarily on CH-π interactions and a few weaker binding modes, making them less effective in binding compared to their aromatic counterparts. Thus, the data from Table 2 and Table 3 demonstrate that diaryl ureas with aromatic R groups possess a much more diverse and energetically favorable interaction profile, significantly improving their binding affinity and potentially their biological activity.

### 2.3. Library of 3D Motifs of Nonbonded Interactions

In order to construct a comprehensive library of three-dimensional (3D) binding motifs to model the DU–protein interactions, we meticulously selected representative motifs of pairwise nonbonded interactions between diaryl ureas and their interacting residues inside the binding pocket. As mentioned above, our large-scale data mining of the PDB resulted in a database of 158 non-redundant high-resolution X-ray crystal structures of DU–protein complexes. Based on this database, 102 unique motifs of nonbonded interaction pairs between diaryl ureas and their interacting residues were extracted. These represent key modes of nonbonded interactions, including 20 hydrogen bonding, 2 salt bridge, 12 cation–π interaction, 24 π–π stacking interactions and 37 CH-π interaction, 3 OH-π interaction, 2 SH-π interaction, 2 NH-π interaction. The selection of these motifs was governed by two main criteria. The first criterion prioritizes proteins that are the targets of disease treatment. The second criterion ensures that these 102 motifs comprehensively cover the diverse spectrum of possible intermolecular interactions, such as hydrogen bonding, salt bridge, and all non-bonded π interactions, in proportion to their occurrence frequencies in the database.

Table S1 provides a full list of all 102 motifs in detail. For easy reference, a motif ID is assigned in column 1. Column 2 defines the mode of nonbonded interactions. Column 3 lists the PDB IDs for the DU-bound complexes from which the binding motifs are extracted. Column 6 lists the interacting pairs, with DUs labeled by the three-letter ligand IDs of the corresponding PDB file and their interacting residues labeled by the residue IDs. Geometrical features for the interacting pairs are given in Columns 4 and 5. For hydrogen bonding pairs, two sets of H-bond angles and distances are listed since there are dual hydrogen bonds in some motifs. For all other modes of nonbonded interactions, the closest atom-to-atom distance between the diaryl ureas and its interacting residue is tabulated. For π–π stacking interactions, the angle is measured between the two π planes of the interacting partners.

#### 2.3.1. Structures 

**Figure A1:**
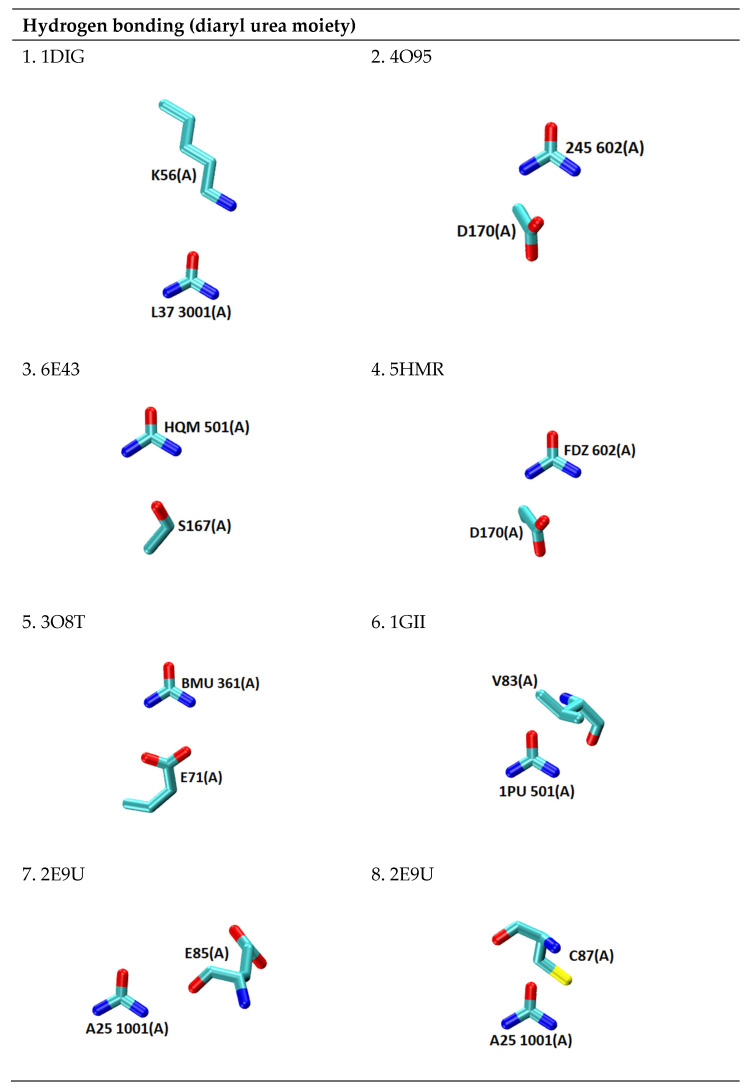

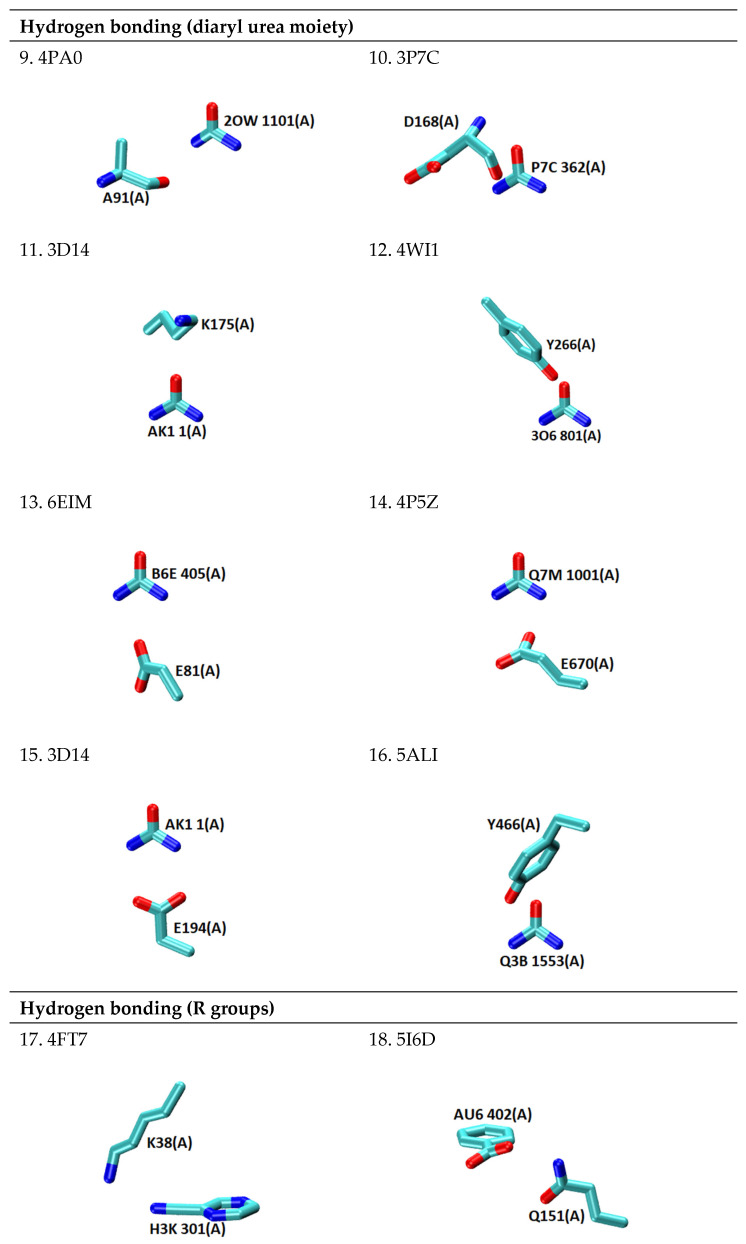

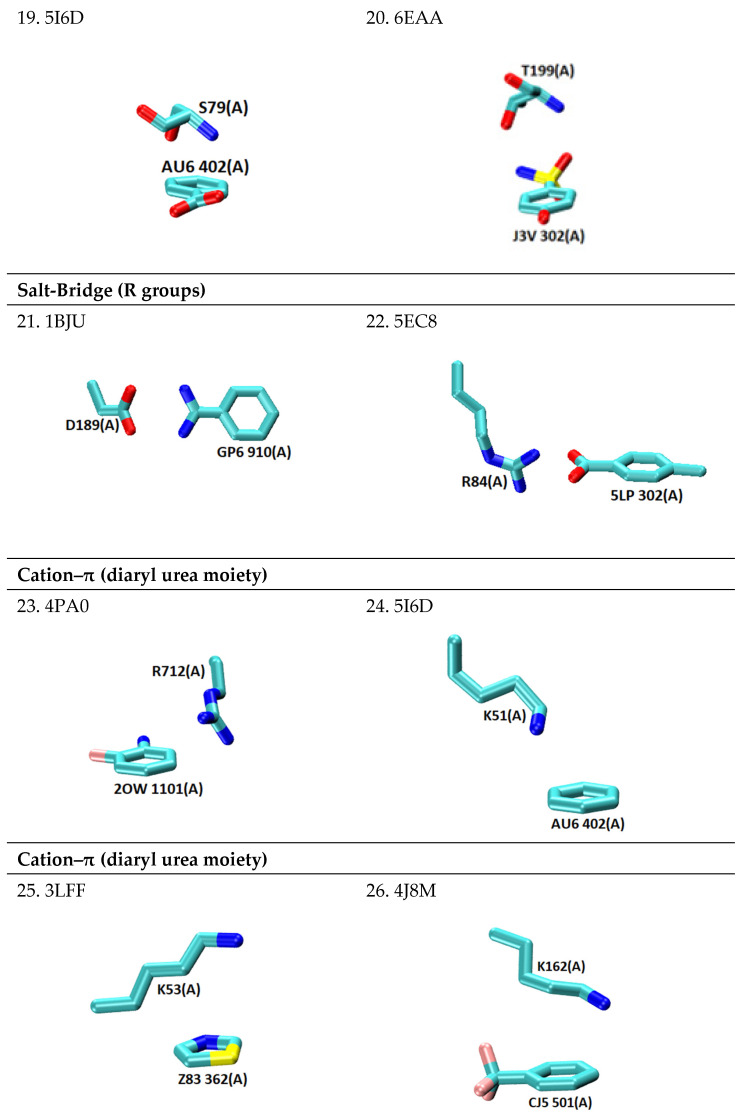

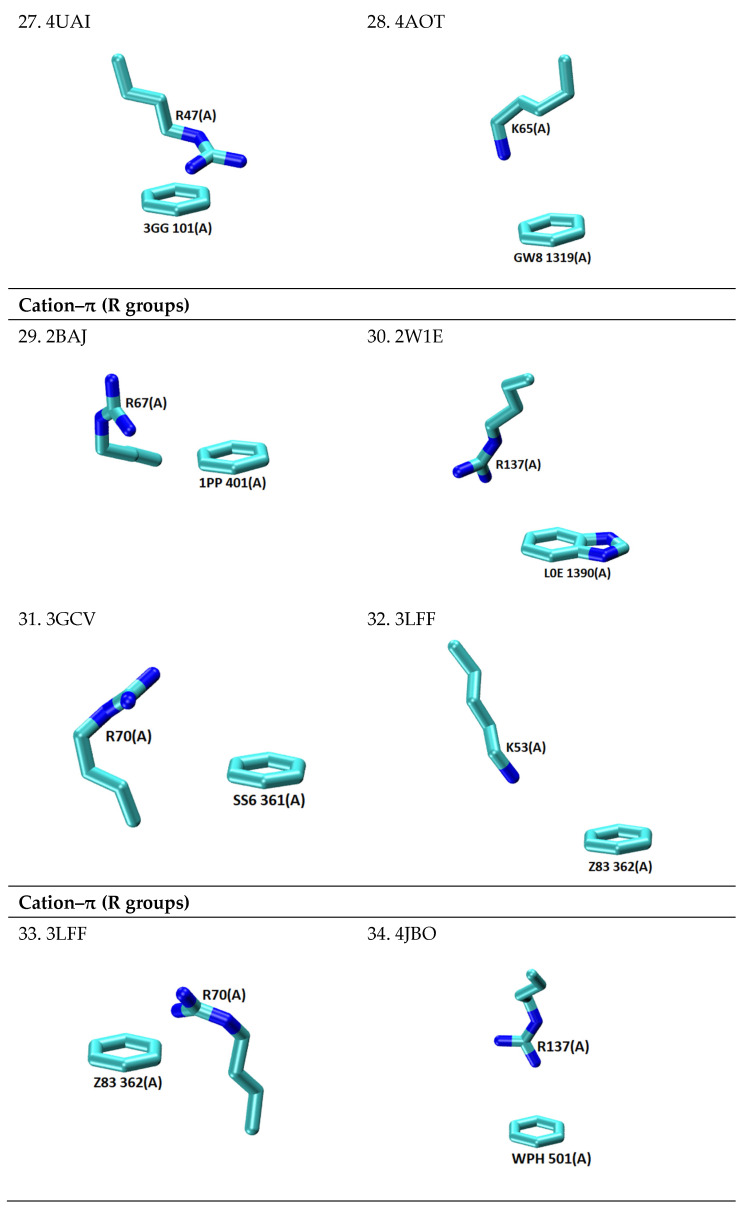

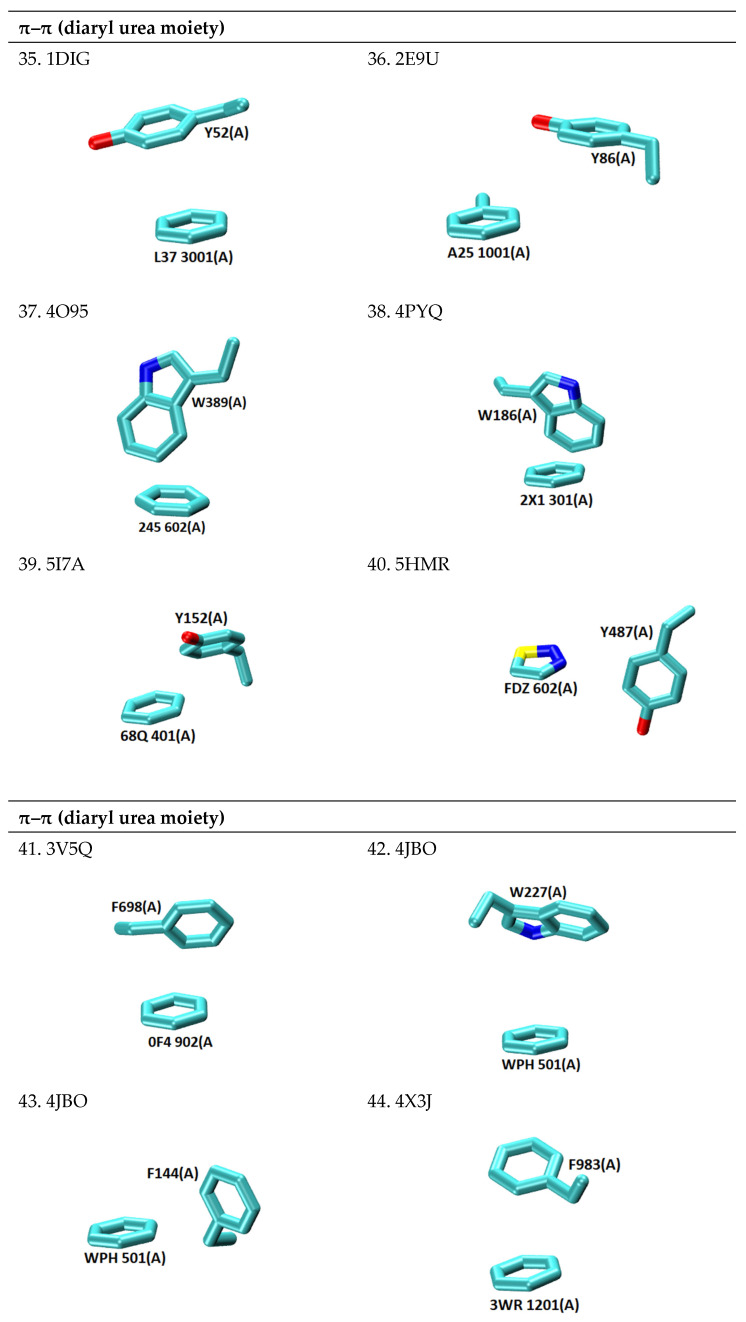

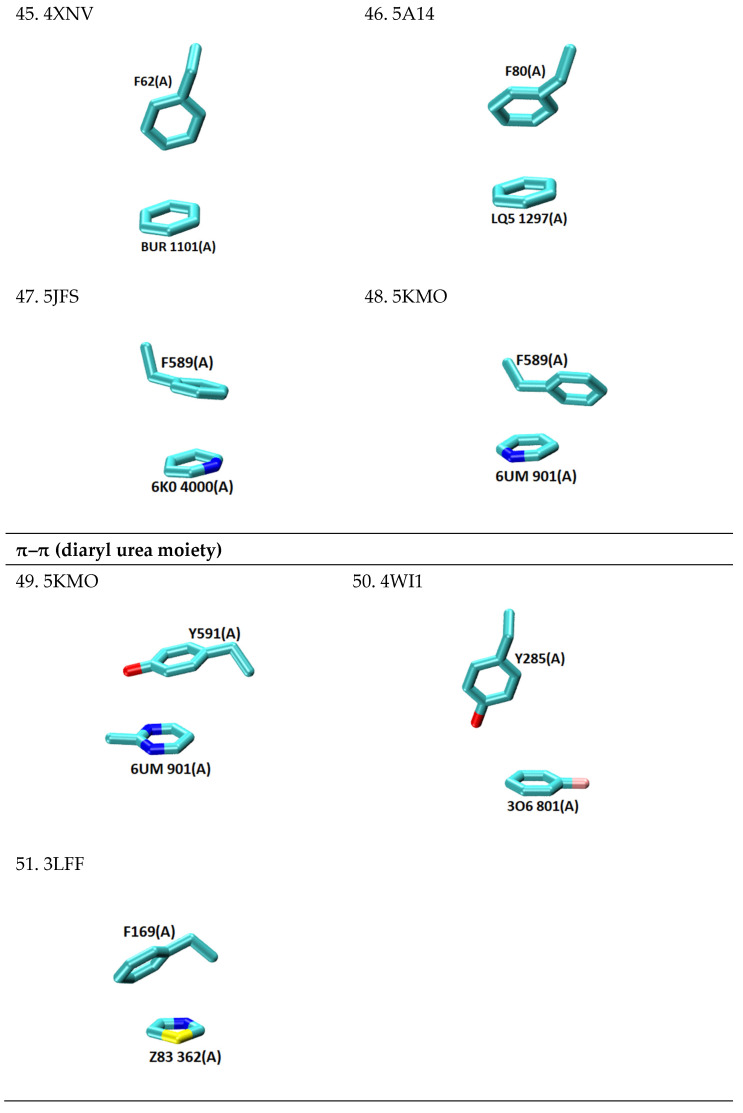

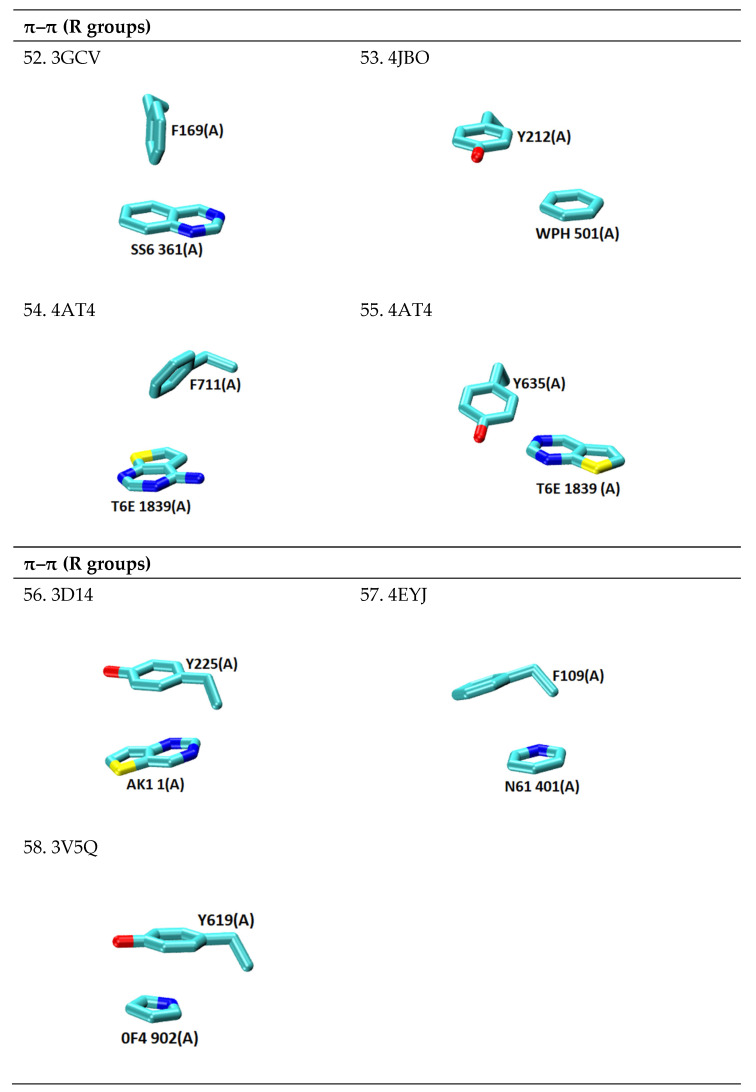

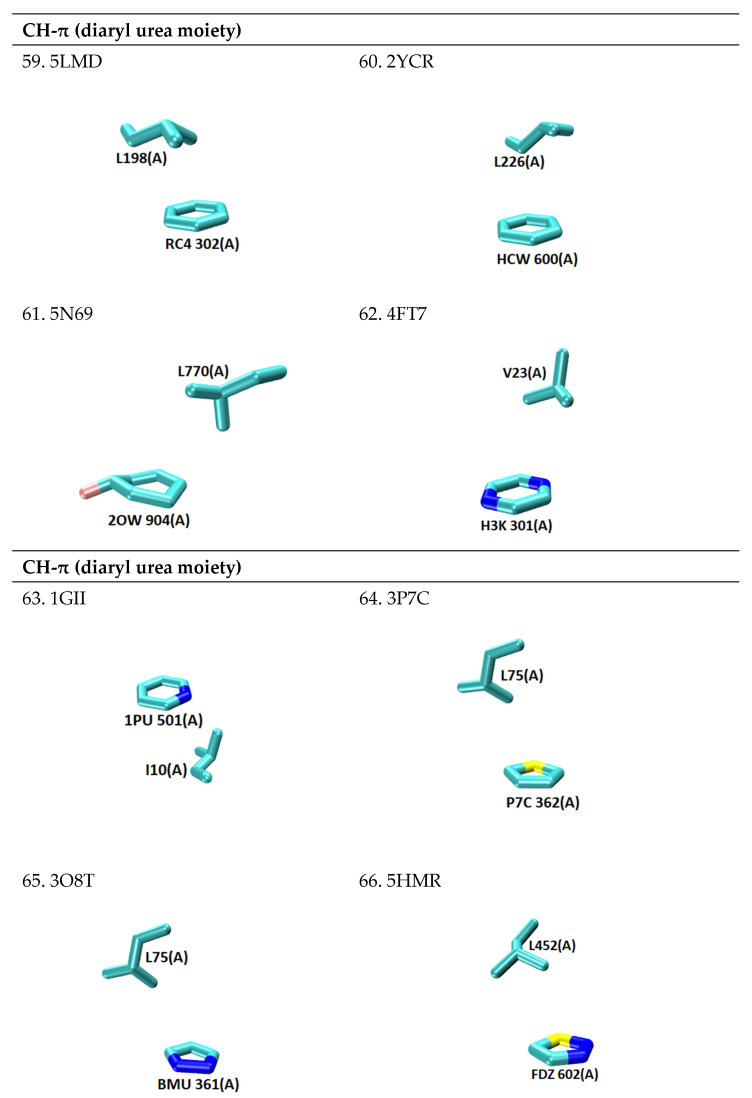

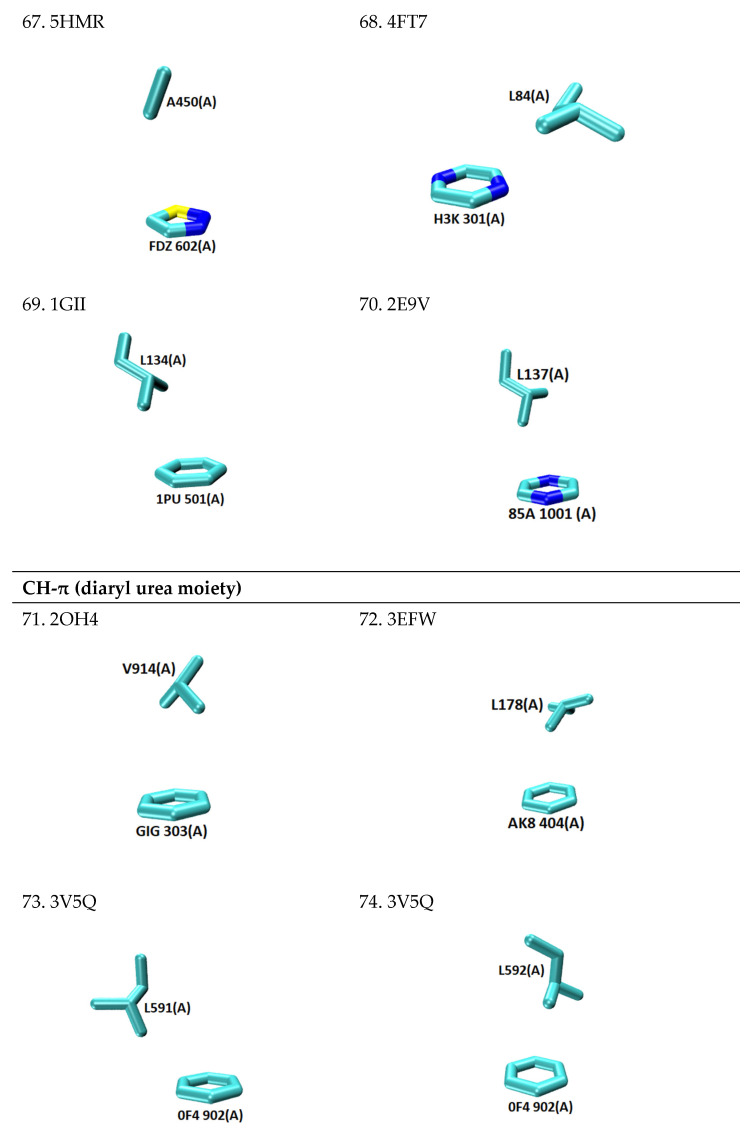

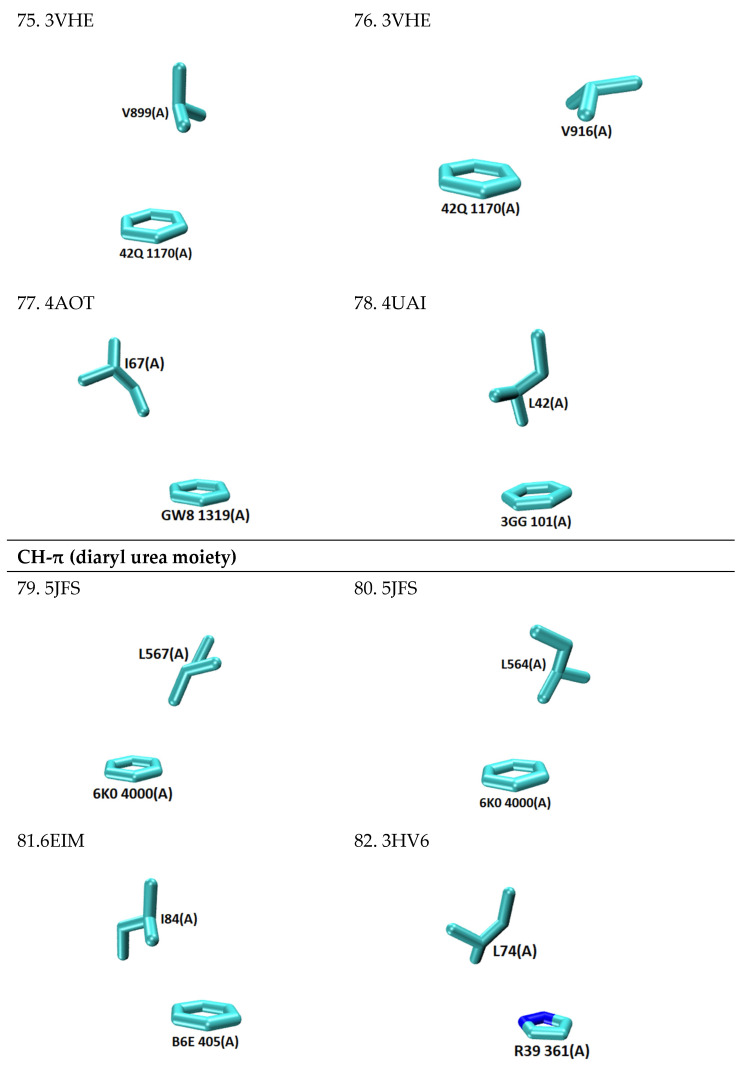

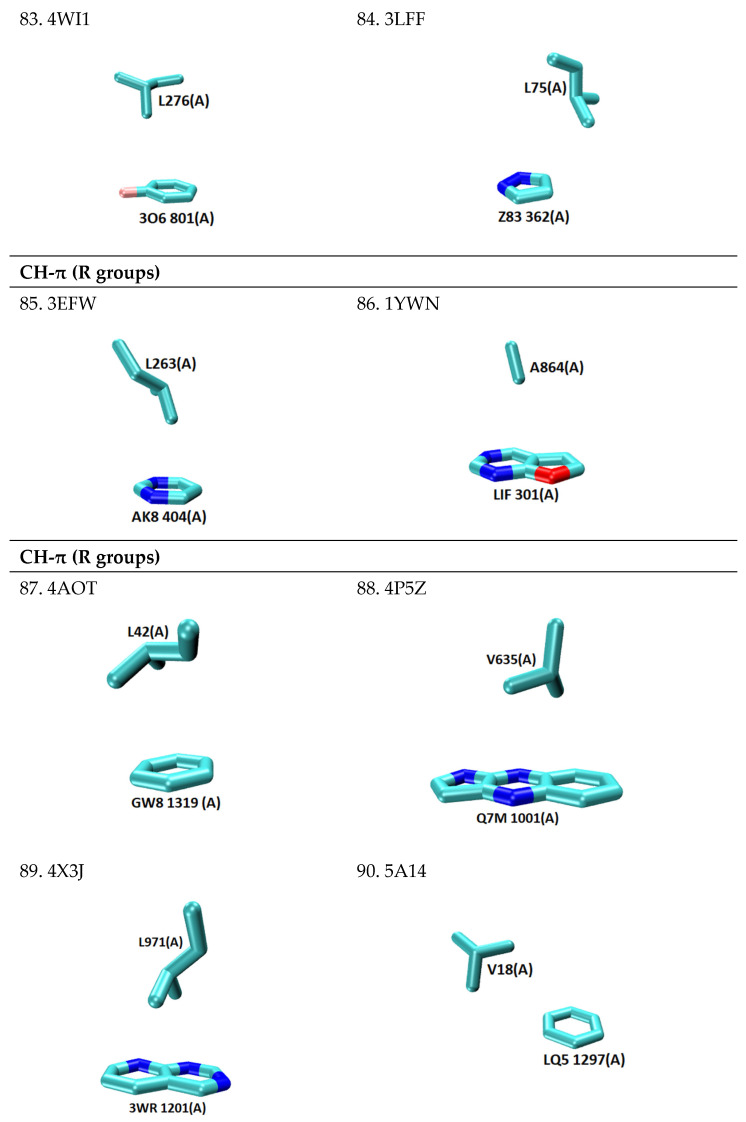

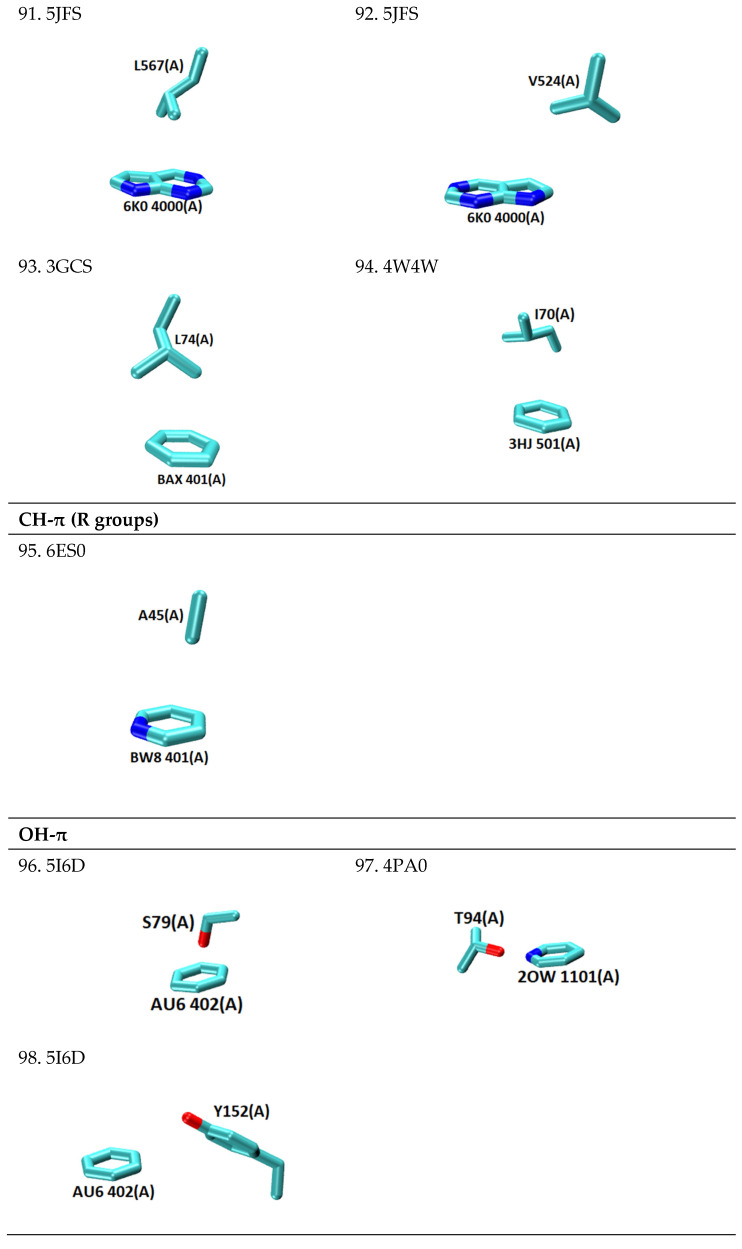

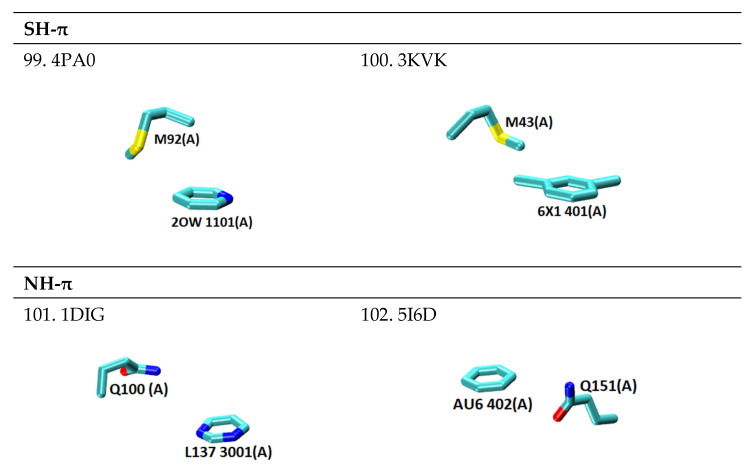
Motifs of nonbonded interactions between diaryl ureas (labeled by the three-letter ligand IDs) and their interacting residues (labeled by the residue IDs) in a licorice representation. Only non-hydrogen atoms are shown. Carbon, oxygen, sulphur and nitrogen atoms are colored in cyan, red, yellow and blue, respectively.

Three-dimensional structures for all 102 motifs of nonbonded interaction pairs between diaryl ureas and their interacting residues are depicted in Figure A1 in the same motif count order as in Table S1. The four-letter codes after the motif count represent the PDB IDs for the DU-bound complexes from which the binding motifs are extracted. For each binding motif, the diaryl ureas and its interacting residue are labeled by the three-letter ligand ID and the residue ID number of the corresponding PDB file, respectively. Given the large size of diaryl ureas molecules, only the functional group of a given diaryl ureas that directly interacts with the residue was kept as part of the 3D motif. The coordinates of all the non-hydrogen atoms were extracted from their respective PDB files. Hydrogen atoms were patched to satisfy the valency by means of a geometry optimization calculation at the HF/6-31+G levels using the Gaussian 09 program.

#### 2.3.2. Energetics 

In order to assess the strength and the relative importance of different types of nonbonded interactions, the strengths of the nonbonded interaction energies for all motifs were evaluated quantum mechanically. The strengths of the intermolecular interaction energies for all 102 motifs were calculated at both the gas phase and the solution phase. The latter aims at a realistic evaluation of the strengths of the intermolecular interactions in the aqueous media where the actual biological interactions occur. Table 4 lists both the gas phase (${∆E}_{int}^{g}$) and the solution phase (${∆E}_{int}^{aq}$) interaction energies for all 102 motifs. The gas phase interaction energies were calculated at the B2PLYP/def2-QZVP level with the basis set superposition error (BSSE) corrections (see Theory and Methods for details). The solution phase interaction energies were obtained indirectly by means of a thermodynamic cycle (see Theory and Methods): ${∆E}_{int}^{aq}={∆E}_{int}^{g}+{∆E}_{Deh}$. The dehydration energy ${∆E}_{Deh}\;$itself was calculated utilizing the SM5.42R solvation model of Cramer and Truhlar.

As shown in Table 4, the calculated solution phase intermolecular interaction energies for the 102 3D motifs are favorable (negative) except cases number 27, 40, and 43. Details on each mode of intermolecular interactions are examined below. All modes of nonbonded interactions associated with 102 motifs are characterized below.

#### 2.3.3. Hydrogen Bonding 

Hydrogen bonding represents a fundamental interaction in DU–protein complexes, playing a critical role in stabilizing these systems. The urea moiety in diaryl ureas acts as both a donor and acceptor, with nitrogen (N) atoms donating and oxygen (O) atoms accepting hydrogen bonds. The gas-phase interaction energies of hydrogen bonds originating from the urea moiety range from −6.0 to −32.0 kcal/mol, with an average of −17.4 kcal/mol. These strong interactions frequently involve acidic or basic residues, such as glutamic acid (Glu) and aspartic acid (Asp), with dual hydrogen bonding often observed. Specifically, the strongest hydrogen bonds are formed between the nitrogen atoms of the urea moiety and the carboxylate oxygens of Asp and Glu. These high interaction energies are largely attributed to the negative charge of the carboxylate groups of these residues. Another notable strong hydrogen bond involves the oxygen of the urea moiety interacting with the nitrogen of lysine’s side chain, with gas-phase energies ranging from −20.8 to −32.0 kcal/mol. In this instance, the positive charge of lysine’s side chain enhances the intermolecular interaction strength. Hydrogen bonds originating from the R groups of diaryl ureas also contribute significantly to binding, with gas-phase interaction energies ranging from −2.4 to −29.7 kcal/mol and an average of −18.8 kcal/mol. These interactions involve the R groups forming hydrogen bonds with surrounding residues, further stabilizing the DU–protein complex.

Considering the physiological environment where binding occurs, solvent effects significantly impact hydrogen bond strengths. Solvation corrections, evaluated using a thermodynamic cycle, indicate a marked reduction in interaction energies in the solution phase. For hydrogen bonds originating from the urea moiety, solution-phase interaction energies range from −0.3 to −2.5 kcal/mol, with an average of −1.6 kcal/mol. For instance, the dual-mode mono-residue Ni-Oi motif has a solution-phase energy of −1.9 kcal/mol, while the hydrogen bond between the oxygen of the urea moiety and the main chain nitrogen of aspartic acid exhibits an interaction energy of −1.7 kcal/mol. Hydrogen bonds originating from R groups show solution-phase energies ranging from −1.4 to −3.5 kcal/mol, averaging −2.4 kcal/mol. The solvation correction energies are positive, with average values of +15.8 kcal/mol and +16.4 kcal/mol for hydrogen bonds from the urea moiety and R groups, respectively. This underscores the necessity of accounting for solvent effects when assessing binding strengths to ensure reliable evaluations of intermolecular interactions in biological systems.

Despite the reductions in binding strength due to solvation, the solution-phase interaction energies remain favorable for all representative hydrogen bonding motifs. These findings highlight the critical contribution of hydrogen bonding, both from the urea moiety and R groups, to the favorable interactions between diaryl ureas and their target proteins.

#### 2.3.4. Salt Bridges

Salt bridges involve electrostatic interactions between oppositely charged groups in diaryl ureas and proteins. Charged R groups in diaryl ureas interact with basic residues, such as lysine (Lys), or acidic residues, such as aspartic acid (Asp), in proteins. These interactions exhibit strong gas-phase interaction energies, with selected representatives displaying values of −115.0 kcal/mol and −106.3 kcal/mol, resulting in an average of −110.7 kcal/mol. However, as expected, solvation corrections significantly diminish the strength of salt bridge interactions. The solution-phase interaction energies average −6.5 kcal/mol, reflecting the substantial dehydration energy required to form salt bridges in an aqueous environment. Overall, the presence of salt bridges, although not a frequent occurrence, enhances the stability of DU–protein complexes.

#### 2.3.5. Cation–π Interaction

Cation–π interactions occur between positively charged residues, such as lysine (Lys) and arginine (Arg), and the π-electron cloud of aromatic rings in diaryl ureas. Cation–π interactions are categorized into two types: those originating from the diaryl urea moiety and those from R groups. The gas-phase interaction energies for diaryl urea moiety range from 0.2 to −11.4 kcal/mol, with an average value of −5.9 kcal/mol. In contrast, gas-phase interaction energies for the R group range from −0.8 to −6.9 kcal/mol, with an average of −3.8 kcal/mol. In some instances, residues form both CH-π and cation–π interactions, further enhancing binding, as observed in motifs 24 to 27 of Table 4. The solution-phase interaction energies for cation–π interactions are slightly reduced compared to the gas phase, averaging −2.2 kcal/mol for diaryl urea moieties and −1.2 kcal/mol for R groups.

#### 2.3.6. π–π Stacking Interactions

π–π stacking interactions occur between the aromatic rings of diaryl ureas and aromatic residues in proteins such as phenylalanine (Phe), tyrosine (Tyr), and tryptophan (Trp). π–π stacking interactions are divided into two types: those originating from the diaryl urea moiety and those formed by R groups. For the diaryl urea moiety, gas-phase interaction energies range from −0.2 to −3.4 kcal/mol, with an average of −1.9 kcal/mol. In contrast, gas-phase interaction energies for R group interactions range from −1.5 to −3.2 kcal/mol, with an average of −2.2 kcal/mol.

The solution-phase interaction energies for π–π stacking interactions are also favorable, with averages of −1.8 kcal/mol for diaryl urea moiety interactions and −1.5 kcal/mol for R group interactions. The configurations of these interactions vary, including parallel-displaced and T-shaped orientations. As expected, solvation corrections have a smaller impact on π–π stacking interactions compared to other types, such as hydrogen bonding or cation–π interactions. These findings underscore the importance of considering π–π stacking interactions as a critical stabilizing factor in DU–protein complexes.

#### 2.3.7. CH-π Interactions

CH–π interactions dominate the interaction profile, largely due to the abundant number of aromatic rings in diaryl ureas and high propensity of their interacting aliphatic residues such as alanine (Ala), leucine (Leu), and isoleucine (Ile) in the binding pockets of DU-binding proteins. The gas-phase interaction energies for CH–π interactions originating from the diaryl urea moiety range from −0.4 to −2.4 kcal/mol, with an average of −1.0 kcal/mol. In contrast, gas-phase interaction energies for R group CH–π interactions range from −0.7 to −2.9 kcal/mol, with an average of −1.6 kcal/mol. The solution-phase interaction energies for CH–π interactions are favorable for all pairwise interactions studied. The averages are −1.0 kcal/mol for interactions originating from the diaryl urea moiety and −2.0 kcal/mol for those from R groups. Notably, the impact of solvation corrections on CH–π interactions is smaller than for π–π stacking or cation–π interactions. Due to both the high frequency and stabilizing nature of CH–π interactions, the CH–π interactions play a critical role in diaryl ureas binding.

#### 2.3.8. XH-π Interaction

XH–π interactions (where X = O, S, N) provide additional stabilization to DU–protein complexes. These include OH–π (Motifs 96–98), SH–π (Motifs 99–100), and NH–π (Motifs 101–102) interactions. Gas-phase interaction energies average −1.2 kcal/mol for OH–π, −1.2 kcal/mol for SH–π, and −0.3 kcal/mol for NH–π. Solution-phase energies are slightly reduced, averaging −0.7 kcal/mol, −0.9 kcal/mol, and −0.7 kcal/mol, respectively. These interactions often accompany other modes of nonbonded interactions, enhancing overall binding stability.

In summary, multiple modes of nonbonded interactions in DU–protein complexes are responsible for molecular recognition of diaryl ureas in proteins. Hydrogen bonding and π-based interactions (π–π, CH–π, cation–π) are the dominant contributors to binding, while salt bridges and XH–π interactions provide additional specificity and stabilization. These findings highlight the potential of diaryl ureas as versatile inhibitors in drug design, with their interaction profiles offering valuable insights for structure-based optimization.

### 2.4. Illustrative Examples of Molecular Recognition of Diaryl Ureas in Proteins

To better understand the binding pockets of diaryl ureas (DUs) within their target proteins, we systematically analyzed the nonbonded interactions responsible for molecular recognition in two representative DU–protein complexes. One representative complex featured DUs with aromatic R groups, while the other involved non-aromatic R groups. The aim was to assess the contributions of these R groups to DU binding in their respective protein targets, as well as to evaluate the role of the diaryl urea core structure itself.

A comprehensive structural and binding mode analysis was performed for these two representative DU–protein complexes. The selected representatives allowed for a direct comparison of the interaction profiles of DUs with aromatic and non-aromatic R groups. Specifically, we analyzed the diaryl urea inhibitors 1-{5-tert-butyl-3-[(5-oxo-1,4-diazepan-1-yl)carbonyl]thiophen-2-yl}-3-naphthalen-1-ylurea (ligand ID: P7B) with non-aromatic R groups, and 4-[4-({[4-chloro-3-(trifluoromethyl)phenyl]carbamoyl}amino)phenoxy]-N-methyl-2-pyridinecarboxamide (ligand ID: BAX) with aromatic R groups. As shown in Table A1, P7B binds to vascular endothelial growth factor receptor 2 (VEGFR2), while BAX, also known as Sorafenib, binds to p38 mitogen-activated protein kinase (MAPK14).

**Figure 3:**
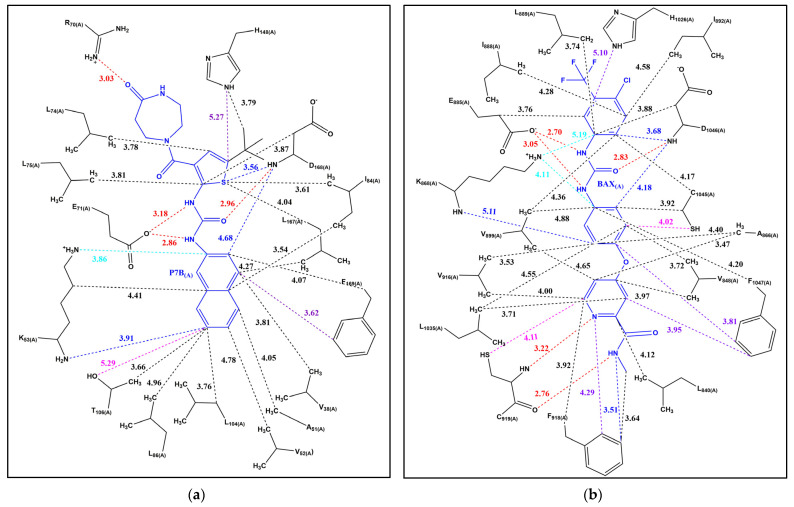
Nonbonded interactions. (**a**) A schematic interaction map between P7B and its interacting residues of mitogen-activated protein kinase 14 (PDB ID: 3P7B); (**b**) A schematic interaction map between BAX and its interacting residues of vascular endothelial growth factor receptor 2 (PDB ID: 3WZE). Dash lines indicate interatomic distance in Å for various intermolecular interactions (color code: hydrogen bonding in red, salt bridges in orange, π–π stacking interactions in purple, CH-π interactions in black, cation–π interactions in cyan, NH-π interactions in blue, OH-π interactions in pink, and SH-π interactions in magenta).

We first inspected the binding pockets of both diaryl ureas inhibitors and identified all nonbonded interactions, including hydrogen bonds, salt bridges, π–π stacking interactions, cation–π interactions, CH-π interactions, and XH-π interactions (XH = NH, OH, SH). Figure 3a provides a schematic map of the intermolecular interactions between P7B and its binding residues in VEGFR2, derived from the 1.90 Å resolution X-ray crystal structure (PDB ID: 3P7B) [27]. This map highlights all modes of interactions, illustrating the critical molecular recognition features of P7B within the VEGFR2 binding pocket. To further contextualize these interactions, Figure S2 depicts the detailed three-dimensional arrangement of residues within 5.6 Å of P7B, offering a comprehensive spatial perspective.

Similarly, Figure 3b depicts the intermolecular interaction map for BAX and its interacting residues in MAPK14, based on the 1.90 Å resolution X-ray crystal structure (PDB ID: 3WZE) [28]. This visualization captures the diversity of interaction modes contributing to the stability of the BAX-MAPK14 complex. Complementing this analysis, Figure S3 displays the three-dimensional arrangement of residues within 5.6 Å of BAX, providing insights into the spatial organization critical for molecular recognition.

Furthermore, modes of nonbonded interactions of P7B and BAX are categorized in Table 5 and Table 6, respectively. The 2nd column lists the interacting residue, with the modes of nonbonded interactions given in column 3. As can be seen from the tables and figures, the number of nonbonded interactions formed by BAX is considerably larger than those formed by P7B, largely due to the existence of the aromatic R group of BAX. Another notable observation is that both diaryl ureas feature dual-mode or even multimode interactions. For example, the oxygen of the urea moiety of BAX forms a hydrogen bond with the main chain nitrogen of ASP 1046, which also forms CH-π and NH-π interactions with the aromatic rings of the diaryl urea moiety.

The strengths of nonbonded intermolecular interactions between the two diaryl ureas and their target protein kinases were quantified using the double hybrid DFT method B2PLYP/def2-QZVP (see Theory and Methods for details). The pairwise intermolecular interaction energies obtained from these calculations are presented in column 4 of Table 5 and Table 6, while column 5 provides a summary based on molecular components. These tables detail the contributions of various molecular components, including the diaryl urea core, the R groups, and their combined effects, to the overall binding energies.

As evident from Figure 3, the predominant mode of interaction across both diaryl urea molecules is nonbonded π interactions. In the case of the diaryl urea with non-aromatic R groups (P7B), the majority of these interactions are formed by the diaryl urea moiety itself, which engages extensively in nonbonded π interactions such as CH–π interactions. The non-aromatic R groups in P7B contribute additional stability by forming CH–π interactions with residue H148 and a hydrogen bond with residue R70. In contrast, for the diaryl urea with aromatic R groups (BAX), both the diaryl urea moiety and the aromatic R groups play significant roles in binding. The aromatic R groups introduce a large number of nonbonded π interactions, which greatly enhance the overall binding strengths.

The energetic analysis reveals that the contributions of nonbonded π interactions between the aromatic moieties of diaryl ureas and aliphatic or aromatic residues far outweigh those of hydrogen bonding interactions. This dominance is evident in Table 5 and Table 6, which show that CH–π interactions between the aromatic moieties of diaryl urea inhibitors and the aliphatic residues of target proteins form the foundation of binding. Moreover, the inclusion of aromatic R groups markedly increases the number and strength of nonbonded π interactions, underscoring their critical role in enhancing the binding affinity. Conversely, diaryl ureas with non-aromatic R groups demonstrate limited contributions from the R groups themselves, relying primarily on the interactions formed by the diaryl urea moiety.

The comparison between P7B and BAX further illustrates the contrasting contributions of non-aromatic and aromatic R groups. For P7B, the binding is largely driven by the interactions formed by the diaryl urea moiety (89.7% of interaction energy), with the non-aromatic R groups providing modest additional stabilization. In contrast, BAX benefits significantly from its aromatic R groups, which introduce a substantial number of nonbonded π interactions that complement the contributions of the diaryl urea moiety.

Hydrogen bonding, while not as dominant as nonbonded π interactions, plays a vital anchoring role for the formation of DU–protein complexes. The oxygen atom of the urea moiety frequently forms hydrogen bonds with the main chain nitrogen of surrounding residues. Notably, a dual hydrogen bonding pattern is commonly observed, wherein the nitrogen atoms of the urea moiety simultaneously interact with the oxygen atoms of either the main chain or the side chain of adjacent residues.

Interestingly, hydrogen bonds are often accompanied by nonbonded π interactions, which further enhance the strength of the interaction. In many cases, aspartic acid residues not only form hydrogen bonds with the oxygen of the urea moiety but also engage in CH–π and NH–π interactions with the aromatic rings of the diaryl urea moiety. The calculated interaction energies for aspartic acid residues in representative examples are −6.5 and −7.3 kcal/mol, which exceed the average energy of hydrogen bonds formed by the urea moiety. This synergy between hydrogen bonding and nonbonded π interactions highlights the potential of aromatic rings in diaryl ureas to significantly bolster overall binding affinity.

In summary, the comparative analysis reveals significant differences in the interaction profiles of diaryl ureas based on the nature of their R groups. Diaryl ureas with aromatic R groups, as exemplified by BAX, exhibit superior molecular recognition capabilities due to their ability to engage in a diverse array of nonbonded interactions, particularly nonbonded π interactions. This contrasts with the non-aromatic R groups in P7B, which rely primarily on hydrogen bonding and van der Waals interactions, resulting in less interaction diversity and reduced binding strength. The findings underscore the critical role of R group aromaticity in enhancing binding affinity and specificity, providing valuable insights for the rational design of diaryl urea-based inhibitors.

## 3. Theory and Methods

### 3.1. Data Mining and Binding Mode Analysis

To investigate the molecular determinants of DU–protein recognition, we performed a large-scale data mining of the Protein Data Bank (PDB, https://www.rcsb.org). Protein–ligand complexes with a resolution of 3.0 Å or better were included to ensure high structural accuracy and comprehensive representation of ligands. To avoid redundancy, proteins with sequence identities exceeding 30% were excluded, leveraging the established correlation between sequence identity and structural similarity [29]. This filtering yielded a dataset comprising 158 non-redundant DU–protein complexes.

The Visual Molecular Dynamics (VMD) program [26] was employed to examine nonbonded interactions, focusing on nonbonded π interactions, hydrogen bonding, and salt bridges. All residues within 5.6 Å of the diaryl ureas molecule were identified and analyzed. Hydrogen bonding was assessed using a donor-to-acceptor cutoff distance of 3.5 Å, while cutoffs of 5.6 Å, 5.0 Å, and 5.5 Å were applied for π–π/cation–π, CH-π, and NH-π/OH-π/S-π interactions, respectively. For cation–π interactions, distances were defined as the proximity between the side-chain nitrogen of lysine or the central carbon atom of the guanidinium group of arginine to the aromatic ring system. Similarly, π–π stacking interactions were quantified as the closest atom-to-atom distance between aromatic systems of diaryl ureas and residues.

Nonbonded interactions were counted as follows. For hydrogen bonding, each interaction involving a single hydrogen atom of diaryl urea with distinct acceptor atoms was counted independently. This criterion was similarly applied to interactions where a protein residue acted as the donor and diaryl urea as the acceptor. For nonbonded π interactions, if one residue interacts with more than one aromatic ring through the same type of nonbonded π interaction, we count the interaction with each aromatic ring as one interaction only. The role and the contribution of diaryl urea moiety and R groups in the molecular recognition of diaryl ureas were then identified.

### 3.2. Quantum Chemical Calculation of Intermolecular Interaction Energies

The crystal structures of all complexes analyzed in this study were obtained from the Protein Data Bank. Atomic coordinates for non-hydrogen atoms of the diaryl ureas inhibitors and their interacting protein residues were extracted directly from the X-ray crystal structures. Hydrogen atoms were patched to all interacting pair models to satisfy the valence and their positions were optimized at the HF/6-31+G* level with the positions of non-hydrogen atoms fixed using the Gaussian 09 software package [30].

For the intermolecular interaction energy calculations, the following scheme for the protein–ligand complex formation in solution was used:

(1) $$A\left(aq\right)\;\;\;\;\;\;+\;\;\;\;\;\;B(aq)\;\;\overset{{∆E}_{int\;}^{aq}}{\to }\;\;AB(aq)\;{∆G}_{P}^{sol}↑\;{\;\;\;\;\;\;\;\;\;\;\;\;∆G}_{L}^{sol}↑\;\;\;\;\;\;\;\;\;\;\;\;\;\;\;↑{∆G}_{PL}^{sol}\;A(g)\;\;\;\;\;\;+\;\;\;\;\;\;\;\;B(g)\;\;\;\;\underset{{∆E}_{int\;}^{g}}{\to }\;\;\;\;AB(g)\;$$

This scheme underpins our analysis of the binding strengths between diaryl urea and its binding residues. Similar schemes have been employed in previous solution-phase binding affinity calculations for multiple ligand–protein complexes [31,32,33]. Upon binding, each monomer (ligand or protein) experiences a partial loss of its solvation shell, incurring a dehydration energy penalty. Consequently, the binding energy in solution (${∆E}_{int\;}^{aq}$) is assessed by correcting gas-phase intermolecular interaction energies (${\Delta E}_{Int}^{g}$) for dehydration energy (${∆E}_{Deh}$) as follows:

(2) $${\Delta E}_{Int}^{aq}={\Delta E}_{Int}^{g}+{∆E}_{Deh}$$

Gas-phase interaction energies were calculated using the supermolecular approach. In the supermolecular approach, the gas phase energy of interaction between the ligand (A) and its binding protein (B) is defined as the difference between the energy of the interacting dimer $E_{AB}$ and the sum of the energies of monomers $E_{A}$ and $E_{B}$.

(3) $${\Delta E}_{Int}^{g}=E_{AB}–(E_{A}+E_{B})$$

The intermolecular interaction energy calculations were performed using the ORCA 4.0 program [34] by means of the B2PLYP double-hybrid functional [23,24] in conjunction with the def2-QZVP basis set [35] (B2PLYP/def2-QZVP). For efficiency, B2-PLYP was implemented with the resolution of identity (RI) approximation for the perturbation step and RIJK [36] for the SCF step. In RIJK, appropriate auxiliary basis sets are used to substitute both Coulomb (J) and exchange integrals as used in the Kohn–Sham/Fock matrix by auxiliary three-center and two-center electron repulsion integrals. The choice of the double hybrid density functional method B2PLYP coupled with the def2-QZVP basis set is based on a systematic benchmark study [17]. It was found that the double-hybrid RIJK RI-B2PLYP functional is one of the best DFT methods for the treatment of nonbonded interactions in terms of both accuracy and computational efficiency in comparison with the highly accurate CCSD(T) method [17].

The atom-pairwise dispersion correction with the Becke–Johnson damping scheme (D3BJ) developed by Grimme et al. was used to include dispersion forces [22]. To mitigate the Basis Set Superposition Error (BSSE), the counterpoise (CP) correction scheme introduced by Boys and Bernardi was applied. Gas-phase interaction energy calculations were performed using the ORCA program [34].

The continuum solvation model SMD model [37] developed by Truhlar and co-workers was used in the calculation of the solvation energies (${∆G}_{i}^{Sol}$, i = AB, A, B) employing the Gaussian 09 software [30]. The dehydration energy ${∆E}_{Deh}$ is thus obtained by the following relationship:

(4) $${∆E}_{Deh}={∆G}_{AB}^{Sol}-{∆G}_{A}^{Sol}-{∆G}_{B}^{Sol}$$

## 4. Conclusions

A multi-faceted computational approach was employed to study the molecular recognition of diaryl ureas in their binding proteins. A large-scale data mining of the Protein Data Bank yielded an in-house database of 158 non-redundant, high-resolution crystal structures of diaryl ureas bound to proteins. Notably, 64.0% of the diaryl ureas studied were derivatized with aromatic R groups. A systematic analysis of nonbonded intermolecular interactions, including hydrogen bonding, salt bridges, π–π stacking, CH-π interactions, NH-π interactions, OH-π interactions, S-π interactions, and cation–π interactions, provided key insights into the molecular recognition underpinning diaryl urea binding.

The binding mode analysis identified CH-π interactions as the most dominant, averaging 15.4 interactions per complex, underscoring their critical role in stabilizing protein–ligand complexes. Hydrogen bonding, facilitated by the urea moiety acting as both a donor and acceptor, emerged as another critical determinant for binding. π–π stacking and cation–π interactions further enhanced stability, complementing the interactions mediated by the urea core. Aromatic R groups were shown to improve binding affinity and specificity by introducing diverse and energetically favorable nonbonded π interactions, as well as additional hydrogen bonds.

To further elucidate the molecular features driving these interactions, a library of 102 representative 3D binding motifs was constructed. This library included 20 hydrogen bonding motifs, 2 salt bridges, 12 cation–π interactions, 24 π–π stacking interactions, 37 CH-π interactions, 3 OH-π interactions, 2 SH-π interactions, and 2 NH-π interactions. Advanced quantum chemical calculations using the B2PLYP method quantified the energetic contributions of these interactions, confirming that hydrogen bonding and nonbonded π interactions (π–π, CH-π, and cation–π) are the primary contributors to binding. Salt bridges and XH-π interactions provided additional specificity and stabilization, emphasizing the diversity of molecular interactions utilized by diaryl ureas.

A comparative analysis between diaryl ureas with aromatic and non-aromatic R groups further highlighted the importance of aromatic derivatization in molecular recognition. Diaryl ureas with aromatic R groups exhibited significantly enhanced binding affinity compared to their non-aromatic counterparts. This improvement was attributed to the introduction of diverse nonbonded π interactions, such as π–π stacking, CH-π interactions, and NH-π interactions, which were either absent or weaker in non-aromatic derivatives. In contrast, diaryl ureas with non-aromatic R groups primarily relied on hydrogen bonding and electrostatic interactions, which, while important, contributed less to binding stability and lacked the spatial flexibility of nonbonded π interactions. Additionally, aromatic R groups expanded the interaction footprint within protein binding pockets, engaging residues inaccessible to non-aromatic derivatives. This broader interaction profile not only increased favorable contacts but also improved binding specificity and affinity, confirming the pivotal role of aromatic R group derivatization in optimizing DU–protein interactions.

In summary, this study highlights the versatility and therapeutic potential of diaryl urea (DU) scaffolds, particularly when functionalized with aromatic R groups. These modifications enhance binding affinity and specificity by facilitating key nonbonded π interactions, including π–π stacking and CH-π interactions, with both aromatic and aliphatic residues. The incorporation of aromatic R groups extends the spatial reach of diaryl ureas within binding pockets, allowing them to engage previously inaccessible residues and strengthen interactions with those already interacting with the urea moiety. This expanded interaction network contributes to the overall stabilization of the protein–ligand complex.

The molecular insights gained from this study establish a mechanistic framework for understanding DU–protein interactions, emphasizing the importance of optimizing structural and energetic profiles for effective inhibitor design. The findings demonstrate that selective inhibition of kinase targets by DUs relies on a synergistic interplay between hydrogen bonding and nonbonded π interactions. The urea core forms directional hydrogen bonds with conserved backbone residues, such as those in the hinge region (e.g., NH/CO groups), while aromatic R groups engage in CH-π interactions (−2.5 to −5.0 kcal/mol) and π–π stacking interactions (−3.0 to −6.0 kcal/mol) within isoform-specific hydrophobic pockets. Notably, 64.6% of analyzed DU–protein complexes contain aromatic R groups, which expand the interaction footprint by accessing sub-pockets rich in aliphatic and aromatic residues. Additionally, quantum chemical calculations (B2PLYP/def2-QZVP) reveal that cation–π interactions with positively charged residues, such as Lys and Arg near ATP-binding sites, further enhance specificity. These insights provide a blueprint for designing diaryl ureas as potent and selective kinase inhibitors with optimized π-rich substituents that exploit the diverse hydrophobic and electrostatic landscapes of kinase active sites, balancing broad anchoring hydrogen bonds with selective π-driven interactions.

Overall, this study reinforces the potential of diaryl ureas as highly adaptable scaffolds for targeted therapy development, particularly in cancer and other protein-mediated diseases.

## Figures and Tables

**Table 1:** The average count of nonbonded interactions in all 158 diaryl ureas–protein complexes.

Type of Interaction	Average Count
Hydrogen bonding	4.0
Cation–π interactions	1.2
π–π interactions	3.0
CH-π interactions	15.4
NH-π interactions	4.3
OH-π interactions	1.1
SH-π interactions	0.8
Salt bridge	0.2
CH-π interactions *	0.8

*: Designate π-system originated from the protein residues.

**Table 2:** The average count of nonbonded interactions formed by diaryl ureas with non-aromatic R groups in the 56 diaryl ureas-protein complexes.

Type of Nonbonded Interaction	% of Occurrence	Average Count
Hydrogen bonding (urea moiety)	63.2	1.6
Cation–π interaction	46.4	0.5
π–π interaction	91.1	2.3
CH-π interaction	100.0	9.3
NH-π interaction	100.0	3.2
OH-π interaction	71.4	1.1
SH-π interaction	25.0	0.3
Hydrogen bonding (R)	71.4	2.1
Salt bridge interaction (R)	23.2	0.3
CH-π interaction * (R)	50.0	0.8

(R): The nonbonded interactions are formed by the R groups of the diaryl ureas. *: Designate π-system originated from the protein residues.

**Table 3:** The average count of nonbonded interactions formed by diaryl ureas with aromatic R groups in 102 diaryl ureas–protein complexes.

Type of Nonbonded Interaction	% of Complexes	Average Count
Hydrogen bonding (urea moiety)	87.3	2.5
Cation–π interaction	77.5	1.0
π–π interaction	85.3	2.0
CH-π interaction	100.0	10.8
NH-π interaction	98.0	2.8
OH-π interaction	43.1	0.7
SH-π interaction	37.6	0.6
Hydrogen bonding (R)	80.4	1.6
Cation–π interaction (R)	38.2	0.6
π–π interaction (R)	74.5	1.4
CH-π interaction (R)	98.0	7.9
NH-π interaction (R)	86.3	2.0
OH-π interaction (R)	43.6	0.5
SH-π interaction (R)	39.6	0.5
Salt bridge interaction (R)	8.9	0.2
CH-π interaction * (R)	52.9	0.8

(R): The nonbonded interactions are formed by the R groups of the diaryl ureas. *: Designate π-system originated from the protein residues.

**Table 4:** Intermolecular interaction energies (in kcal/mol) calculated at the B2PLYP level for the 3D binding motifs of diaryl ureas in proteins.

No.	Interaction Mode	PDB ID	Residue		${\Delta E}_{int}^{g}$ (kcal/mol)	${\Delta E}_{Deh}$ (kcal/mol)	${\Delta E}_{int}^{aq}$ (kcal/mol)
1	H-Bonding (Diaryl Urea)	1DIG	K56		−20.8	19.5	−1.3
2		4O95	D170		−25.6	24.0	−1.6
3		6E43	S167		−6.3	5.8	−0.5
4		5HMR	D170		−25.6	24.2	−1.4
5		3O8T	E71		−31.4	29.3	−2.1
6		1GII	V83		−13.8	11.9	−1.9
7		2E9U	E85		−8.3	6.8	−1.5
8		2E9U	C87		−7.7	5.5	−2.2
9		4PA0	A91		−6.1	5.7	−0.4
10		3P7C	D168		−10.5	8.8	−1.7
11		3D14	K175		−25.0	23.2	−1.8
12		4WI1	Y266		−6.0	3.5	−2.5
13		6EIM	E81		−27.1	25.3	−1.8
14		4P5Z	E670		−23.7	23.4	−0.3
15		3D14	E194		−32.0	29.5	−2.5
16		5ALI	Y466		−7.9	5.8	−2.1
				**Average**	**−17.4**	**15.8**	**−1.6**
17	H-Bonding (R groups)	4FT7	K38		−18.9	16.1	−2.8
18		5I6D	Q151		−12.6	9.1	−3.5
19		5I6D	S79		−29.7	28.3	−1.4
20		6EEA	T199		−14.0	11.9	−2.1
				**Average**	**−18.8**	**16.4**	**−2.4**
21	Salt bridge (R groups)	1BJU	D189		−115.0	106.0	−9.0
22		5EC8	R84		−106.3	102.3	−4.0
				**Average**	**−110.7**	**104.2**	**−6.5**
23	Cation–π (Diaryl Urea)	4PA0	R712		0.2	−1.7	−1.5
24		5I6D	K51		−11.4	8.5	−2.9
25		3LFF	K53		−5.9	5.2	−0.7
26		4J8M	K162		−8.1	3.8	−4.3
27		4UAI	R47		−3.5	4.3	0.8
28		4AOT	K65		−6.5	2.0	−4.5
				**Average**	**−5.9**	**3.7**	**−2.2**
29	Cation–π (R groups)	2BAJ	R67		−2.3	0.8	−1.5
30		2W1E	R137		−4.9	3.8	−1.1
31		3GCV	R70		−0.8	0.4	−0.4
32		3LFF	K53		−2.5	2.1	−0.4
33		3LFF	R70		−5.5	3.5	−2.0
34		4JBO	R137		−6.9	5.5	−1.4
				**Average**	**−3.8**	**2.6**	**−1.2**
35	π–π (Diaryl Urea)	1DIG	Y52		−2.3	−0.4	−2.7
36		2E9U	Y86		−1.8	0.7	−1.1
37		4O95	W389		−1.2	−0.1	−1.3
38		4PYQ	W196		−3.4	0.6	−2.8
39		5I7A	Y152		−1.1	−0.2	−1.3
40		5HMR	Y487		−1.7	1.7	0.0
41		3V5Q	F698		−3.3	0.2	−3.1
42		4JBO	W227		−1.1	0.2	−0.9
43		4JBO	F144		−0.2	0.2	0.0
44		4X3J	F983		−2.4	−0.2	−2.6
45		4XNV	F62		−1.8	0.0	−1.8
46		5A14	F80		−0.9	−0.8	−1.7
47		5JFS	F589		−3.1	0.0	−3.1
48		5KMO	F589		−2.8	0.0	−2.8
49		5KMO	Y591		−2.9	1.8	−1.1
50		4WI1	Y285		−1.3	−0.2	−1.5
51		3LFF	F169		−1.8	−0.3	−2.1
				**Average**	**−1.9**	**0.2**	**−1.8**
52	π–π (R groups)	3GCV	F169		−2.6	−0.3	−2.9
53		4JBO	Y212		−1.8	0.7	−1.1
54		4AT4	F711		−2.3	−0.1	−2.4
55		4AT4	Y635		−3.2	2.0	−1.1
56		3D14	Y225		−1.8	0.9	−0.9
57		4EYJ	F109		−1.5	1.3	−0.2
58		3V5Q	Y619		−2.2	0.6	−1.7
				**Average**	**−2.2**	**0.7**	**−1.5**
59	CH-π (Diaryl Urea)	5LMD	L198		−2.4	0.0	−2.4
60		2YCR	L226		−1.5	0.0	−1.5
61		5N69	L770		−0.5	−0.4	−0.9
62		4FT7	V23		−1.2	0.6	−0.5
63		1GII	I10		−0.8	0.5	−0.3
64		3P7C	L75		−0.9	−0.2	−1.1
65		3O8T	L75		−0.7	−0.3	−1.0
66		5HMR	L452		−1.0	0.0	−1.0
67		5HMR	A450		−0.4	0.1	−0.3
68		4FT7	L84		−1.4	1.0	−0.4
69		1GII	L134		−0.5	−0.2	−0.7
70		2E9V	L137		−0.8	0.1	−0.7
71		2OH4	V914		−1.3	0.1	−1.2
72		3EFW	L178		−1.6	0.0	−1.6
73		3V5Q	L591		−0.5	−0.2	−0.7
74		3V5Q	L592		−1.8	0.0	−1.8
75		3VHE	V899		−0.9	0.2	−0.7
76		3VHE	V916		−1.1	0.0	−1.1
77		4AOT	I67		−0.5	0.0	−0.5
78		4UAI	L42		−1.4	−0.2	−1.6
79		5JFS	L567		−1.0	−0.2	−1.2
80		5JFS	L564		−0.6	−0.3	−0.9
81		6EIM	I84		−1.1	−0.2	−1.3
82		3HV6	L74		−0.8	0.2	−0.6
83		4WI1	L276		−0.6	0.1	−0.5
84		3LFF	L75		−0.9	−0.2	−1.1
				**Average**	**−1.0**	**−0.1**	**−1.0**
85	CH-π (R groups)	3EFW	L263		−1.4	−0.8	−2.2
86		1YWN	A864		−1.3	−0.2	−1.5
87		4AOT	L42		−1.8	0.1	−1.7
88		4P5Z	V635		−2.9	−0.1	−3.0
89		4X3J	L971		−1.5	−1.2	−2.7
90		5A14	V18		−1.1	−0.1	−1.2
91		5JFS	L657		−2.7	−0.5	−3.2
92		5JFS	A524		−1.1	−0.2	−1.3
93		3GCS	V74		−1.9	−0.3	−2.2
94		4W4W	I70		−1.7	−0.1	−1.8
95		6ES0	A54		−0.7	−0.6	−1.3
				**Average**	**−1.6**	**−0.4**	**−2.0**
96	OH-π	5I6D	S79		−1.5	1.2	−0.3
97		4PA0	T200		−0.7	0.5	−0.2
98		5I6D	Y152		−1.6	−0.1	−1.7
				**Average**	**−1.2**	**0.6**	**−0.7**
99	S-π	4PA0	M92		−1.6	−0.3	−1.9
100		3KVK	M42		−0.2	−0.3	−0.5
				**Average**	**−0.9**	**−0.3**	**−1.2**
101	NH-π	1DIG	Q100		−0.1	−0.1	−0.2
102		5I6D	Q151		−0.4	−0.8	−1.2
				**Average**	**−0.3**	**−0.5**	**−0.7**

**Table 5:** Contributions of different groups of the diaryl urea molecule to the nonbonded interaction energy between P7B and residues of mitogen-activated protein kinase 14.

Group	Residue	Interaction Mode	${\Delta E}_{int}^{aq}$ (kcal/mol)	Total E (kcal/mol)	% Contribution
Diaryl urea	E71	HB, HB	−6.2	−39.9	89.7
	D168	HB, CH-π, NH-π, NH-π	−7.3		
	K53	Cation–π, CH-π, NH-π	−5.6		
	F169	π–π, CH-π	−2.6		
	V38	CH-π	−1.6		
	I84	CH-π, CH-π	−3.8		
	L74	CH-π	−2.2		
	L75	CH-π, CH-π	−3.5		
	L167	CH-π, CH-π	−2.8		
	A51	CH-π	−0.8		
	T106	CH-π, OH-π	−1.5		
	V52	CH-π	−0.2		
	L86	CH-π	−0.3		
	L104	CH-π	−1.3		
R groups	R70	HB	−3.2	−3.2	7.2
Diaryl urea and R groups	H148	π–π, CH-π *	−1.3	−1.3	2.9

*: Designate π-system originated from the protein residues.

**Table 6:** Contributions of different groups of the diaryl urea molecule to the nonbonded interaction energy between BAX and residues of vascular endothelial growth factor receptor 2.

Group	Residue	Interaction Mode	${\Delta E}_{int}^{aq}$ (kcal/mol)	Total E (kcal/mol)	% Contribution
**Diaryl urea**	E885	HB, HB, CH-π	−4.6	−30.2	48.5
	D1046	HB, CH-π, NH-π, NH-π	−6.5		
	K868	Cation–π, Cation–π, CH-π, NH-π	−5.0		
	I892	CH-π	−4.3		
	L889	CH-π	−2.5		
	I888	CH-π	−1.3		
	H1026	π–π	−1.4		
	C1045	CH-π, CH-π, SH-π	−4.6		
**R groups**	C919	HB, HB, NH-π, SH-π	−5.3	−11.8	18.9
	F918	π–π, CH-π, CH-π *, NH-π *	−3.6		
	L840	CH-π	−2.9		
**Diaryl urea and R groups**	F1047	π–π, π–π, CH-π	−7.5	−20.3	32.6
	L1035	CH-π, CH-π	−3.6		
	V899	CH-π, CH-π	−1.9		
	V848	CH-π, CH-π	−2.2		
	V916	CH-π, CH-π	−2.6		
	A866	CH-π, CH-π	−2.5		

*: Designate π-system originated from the protein residues.

## Data Availability

The original data presented in the study are openly available in Protein Databank (www.rcsb.org).

## Supplementary Materials

The following supporting information can be downloaded at: https://www.mdpi.com/article/10.3390/molecules30051007/s1, Figure S1: Chemical structures of the 150 diaryl ureas inhibitors. Table S1: List of representative interacting pairs in diaryl ureas bound protein complexes. Figure S2: Three-dimensional arrangement of P7B and its interacting residues of mitogen-activated protein kinase 14. Figure S3: Three-dimensional arrangement of BAX (sorafenib) and its interacting residues of vascular endothelial growth factor receptor 2.

## Appendix A

**Table A1:** List of diaryl urea binding protein complexes, including ligand identifiers, protein targets, PDB IDs, resolution (Res), and binding affinity parameters (Ki, Kd, IC50).

Ligand	Protein	PDB ID	Res	K_i_ (nM)	K_d_ (nM)	IC_50_ (nM)
FXE	3-oxoacyl-(acyl-carrier-protein) reductase FabG	4BO0	2.4	-	-	200
36K	3-oxoacyl-(acyl-carrier-protein) reductase FabG	4BO2	1.9	-	-	50
34X	3-oxoacyl-(acyl-carrier-protein) reductase FabG	4BO8	2.7	-	-	120
Q7U	3-oxoacyl-(acyl-carrier-protein) reductase FabG	4BNV	2.5	-	-	30
729	ALK tyrosine kinase receptor	5IUG	1.93	-	2.4	177
34Y	ALK tyrosine kinase receptor	5IUH	2.1	-	-	402
3WR	Angiopoietin-1 receptor	4X3J	2.5	-	-	-
919	Angiopoietin-1 receptor	6MWE	2.05	-	-	-
SVR	Arabidopsis thaliana At3g22680	3GAN	2.0	-	-	-
CJ5	Aurora kinase A	4J8M	1.85	-	-	48
WPH	Aurora kinase A	4JBO	2.49	-	-	24
YPH	Aurora Kinase A	4JBP	2.45	-	-	41
T6E	BDNF/NT-3 growth factors receptor	4AT4	2.36	-	-	12
GP6	Beta-trypsin	1BJU	1.8	6200, 16,000	-	-
GP8	Beta-trypsin	1BJV	1.8	2900, 13,000	-	-
Q3B	Bifunctional epoxide hydrolase 2	5ALI	1.85	-	-	86
3H8	c-Jun N-terminal kinase 3	4W4V	2.01	-	-	98, 866, 2836
3HJ	c-Jun N-terminal kinase 3	4W4W	1.9	-	-	6630
3HN	c-Jun N-terminal kinase 3	4W4X	2.65	-	-	-
3HQ	c-Jun N-terminal kinase 3	4W4Y	2.3	-	-	3034
D3B	Carbonic anhydrase 1	6FAF	1.99	306	-	-
EON	Carbonic anhydrase 1	6FAG	1.79	255	-	-
P9B	Carbonic anhydrase 2	3N0N	1.5	50	-	200
6LU	Carbonic anhydrase 2	5JN7	1.52	1765	-	-
J3V	Carbonic anhydrase 2	6EEA	1.63	200	-	-
J6V	Carbonic anhydrase 2	6EEO	1.72	370	-	-
RC4	Carbonic anhydrase 2	5LMD	1.7	-	-	-
J4D	Carbonic anhydrase 2	6EEH	1.63	290	-	-
AYX	Carbonic anhydrase 2	3N2P	1.65	15	-	-
WWV	Carbonic anhydrase 2	3N3J	1.5	3.3	-	-
WWZ	Carbonic anhydrase 2	3N4B	1.6	96	-	-
2X1	Catechol O-methyltransferase	4PYQ	1.39	-	-	4400
1PU	Cell division protein kinase 2	1GII	2.0	-	-	78, 440
2PU	Cell division protein kinase 2	1GIJ	2.2	-	-	-
SVR	Chromobox homolog 7	4X3U	1.63	-	-	8100
LQ5	Cyclin-dependent kinase 2	5A14	2.0	-	9.7, 53	800, 10,000
245	Cytokinin dehydrogenase 4	4O95	1.75	-	-	-
FDZ	Cytokinin dehydrogenase 4	5HMR	2.0	-	-	35,000
EDZ	Cytokinin dehydrogenase 4	5HQX	2.05	-	-	120,000
6X1	Dihydroorotate dehydrogenase, mitochondrial	3KVK	2.05	-	-	700
0BU	E3 ubiquitin-protein ligase XIAP	4MTZ	2.1	-	-	-
Q7M	Ephrin type-A receptor 3	4P5Z	2.0	-	3.9, 39.3	-
B96	Ephrin type-A receptor 3	4TWN	1.71	-	580, 880	1000, 1200
NSC	Flavivirus methyltransferase	5CUQ	1.7	-	-	7700
K9Y	Focal adhesion kinase 1	4K9Y	2.0	-	111	266, 750
KAO	Focal adhesion kinase 1	4KAO	2.39	-	770	7000, 10,000
JHJ	Galactokinase	6Q90	2.4	-	-	-
5KY	Genome polyprotein	5E9Q	1.79	-	-	-
5LP	Genome polyprotein	5EC8	1.71	-	-	-
25D	Glycogen phosphorylase, liver form	3DD1	2.57	-	-	10, 350
26B	Glycogen phosphorylase, liver form	3DDS	1.8	-		139, 506
55	Glycogen phosphorylase, liver form	3DDW	1.9	-	-	1067
6K0	High affinity nerve growth factor receptor	5JFS	2.07	-	-	2
6UJ	High affinity nerve growth factor receptor	5KMM	2.12	-	-	4100
6UM	High affinity nerve growth factor receptor	5KMO	2.67	-	-	4000
SVR	Human alpha-thrombin	2H9T	2.4	-	-	20,000
4R5	Human Dihydroorotate Dehydrogenase	4ZMG	1.9	-	-	-
BUR	Human P2Y1 receptor	4XNV	2.2	6, 61	-	29, 2100
C4V	Indoleamine 2,3-dioxygenase 1	6AZV	2.76	-	-	-
HQM	Indoleamine 2,3-dioxygenase 1	6E43	1.71	-	-	-
351	Kinase domain of insulin receptor	3ETA	2.6	-	-	14
NSC	Lethal factor	1PWP	2.9	500	-	3200
J7J	Maltose/maltodextrin-binding periplasmic protein, Probable global transcription activator SNF2L2	6EG2	2.98	-	-	-
J7G	Maltose/maltodextrin-binding periplasmic protein, Probable global transcription activator SNF2L2	6EG3	2.84	-	-	-
L37	Methylenetetrahydrofolate dehydrogenase/cyclohydrolase	1DIG	2.2	431,000	-	-
1K9	Mitochondrial isocitrate dehydrogenase	4JA8	1.5	-	-	60, 10,000
JK1	Mitogen activated protein kinase 10	3FI2	2.28	-	-	25, 1200
3NL	Mitogen-activated protein kinase 10	4WHZ	1.79	-	-	1, 528
N61	Mitogen-activated protein kinase 13	4EYJ	2.1	-	-	82.72, 620
N58	Mitogen-activated protein kinase 13	5EKN	2.59	-	-	-
Z87	Mitogen-activated protein kinase 14	3LFB	2.6	-	990	-
Z86	Mitogen-activated protein kinase 14	3LFC	2.8	-	287	-
Z84	Mitogen-activated protein kinase 14	3LFE	2.3	-	586	-
Z83	Mitogen-activated protein kinase 14	3LFF	1.5	-	196	135
DG7	Mitogen-activated protein kinase 14	3PG3	2.0	-	1656	3641
L09	Mitogen-activated protein kinase 14	1WBN	2.4	-	-	350
1PP	Mitogen-activated protein kinase 14	2BAJ	2.25	-	4	30
G2G	Mitogen-activated protein kinase 14	2PUU	2.5	-	-	-
1BU	Mitogen-activated protein kinase 14	3GCQ	2.0	-	324	800, 1400
BAX	Mitogen-activated protein kinase 14	3GCS	2.1	-	56, 10,000	37, 3200
R48	Mitogen-activated protein kinase 14	3GCU	2.1	-	156	370, 460
SS6	Mitogen-activated protein kinase 14	3GCV	2.3	-	74	320, 420
B10	Mitogen-activated protein kinase 14	3GI3	2.4	-	1.8	-
R49	Mitogen-activated protein kinase 14	3HV3	2.0	-	-	470, 900
L51	Mitogen-activated protein kinase 14	3HV4	2.6	-	-	290, 310
R24	Mitogen-activated protein kinase 14	3HV5	2.25	-	-	95, 150
R39	Mitogen-activated protein kinase 14	3HV6	1.95	-	300	2300, 5500
1AU	Mitogen-activated protein kinase 14	3HV7	2.4	-	12	90, 170
HIZ	Mitogen-activated protein kinase 14	3IW8	2.0	-	13,400	-
437	Mitogen-activated protein kinase 14	3NNV	2.1	-	-	27
BMU	Mitogen-activated protein kinase 14	3O8T	2.0	-	1160, 1190	784, 21,000
P5K	Mitogen-activated protein kinase 14	3P5K	2.09	-	-	76
P78	Mitogen-activated protein kinase 14	3P78	2.3	-	-	110
P79	Mitogen-activated protein kinase 14	3P79	2.1	-	-	2300
P7A	Mitogen-activated protein kinase 14	3P7A	2.31	-	-	520
P7B	Mitogen-activated protein kinase 14	3P7B	1.9	-	-	18
P7C	Mitogen-activated protein kinase 14	3P7C	2.3	-	-	22
06F	Mitogen-activated protein kinase 14	3UVR	2.1	-	-	-
6UX	Mixed lineage kinase domain-like protein	5KNJ	2.88	-	-	-
CW5	Mucosa-associated lymphoid tissue lymphoma translocation protein 1	6F7I	2.43	-	-	-
2OW	Myosin light chain 3	5N69	2.45	-	-	290
2OW	Myosin-7	4PA0	2.25	100	-	-
SVR	NAD-dependent deacetylase sirtuin-5	2NYR	2.06	-	-	2000, 46,600
0F4	NT-3 growth factor receptor	3V5Q	2.1	-	-	40
AU6	O-phosphoserine sulfhydrylase	5I6D	1.64	-	8000	-
68Q	O-phosphoserine sulfhydrylase	5I7A	2.08	-	320	-
68V	O-phosphoserine sulfhydrylase	5I7H	2.57	-	-	-
A16	O-phosphoserine sulfhydrylase	5I7O	2.49	-	3400	-
68W	O-phosphoserine sulfhydrylase	5I7R	1.73	-	1700	-
6EC	O-phosphoserine sulfhydrylase	5IW8	2.04	-	2200	-
6EQ	O-phosphoserine sulfhydrylase	5IWC	2.7	-	-	-
L64	Phosphatidylinositol-4,5-bisphosphate 3-kinase catalytic subunit gamma isoform	3IBE	2.79	-	-	-
SVR	Phospholipase A2 homolog 2	1Y4L	1.7	-	-	-
3O6	Proline--tRNA ligase	4WI1	1.65	-	-	5000
B96	Protein tyrosine kinase 2 beta	3FZS	1.75	-	990	400, 11,500
0YJ	Protein tyrosine kinase 2 beta	4H1M	1.99	-	-	78, 550
0YH	Protein-tyrosine kinase 2-beta	4H1J	2.0	-	-	1200
KIN	Proto-oncogene tyrosine-protein kinase ABL1	2HZN	2.7	-	-	41
PD3	Proto-oncogene tyrosine-protein kinase Src	3EL7	2.8	-	-	480
PD5	Proto-oncogene tyrosine-protein kinase Src	3EL8	2.3	-	-	25
1AW	Proto-oncogene tyrosine-protein kinase Src	3F3U	2.5	-	18,000	-
AQM	Proto-oncogene tyrosine-protein kinase Src	3TZ8	2.7	-	-	1.1
6G3	Proto-oncogene tyrosine-protein kinase Src	5J5S	2.15	-	-	-
Q1A	Receptor-interacting serine/threonine-protein kinase 2	4NEU	2.57	-	-	10, 630
SR8	Receptor-interacting serine/threonine-protein kinase 2	5AR7	2.71	-	-	63, 320
BW8	Receptor-interacting serine/threonine-protein kinase 2	6ES0	2.38	-	-	2260, 4870
5O0	RNA-directed RNA polymerase NS5	5EHG	2.02	-	-	-
5O3	RNA-directed RNA polymerase NS5	5EHI	1.3	-	-	-
L0E	Serine/threonine kinase 6	2W1E	2.93	-	-	12
AK1	Serine/threonine kinase 6	3D14	1.9	-	-	22
AK2	Serine/threonine kinase 6	3D15	2.3	-	-	9
AK3	Serine/threonine kinase 6	3D2I	2.9	-	-	-
AK4	Serine/threonine kinase 6	3D2K	2.5	-	-	-
AK7	Serine/threonine kinase 6	3DJ7	2.8	-	-	-
GW8	Serine/threonine-protein kinase 10	4AOT	2.33	-	-	-
B6E	Serine/threonine-protein kinase 10	6EIM	1.43	-	-	-
AK8	Serine/threonine-protein kinase 6	3EFW	2.29	-	-	4
AKI	Serine/threonine-protein kinase 6	3M11	2.75	-	-	43
77A	Serine/threonine-protein kinase CHK1	2E9P	2.6	20	-	22
A25	Serine/threonine-protein kinase CHK1	2E9U	2.0	7.94	-	10
85A	Serine/threonine-protein kinase CHK1	2E9V	2.0	12.59, 13	-	13
H1K	Serine/threonine-protein kinase CHK1	4FT3	2.5	631	-	-
H2K	Serine/threonine-protein kinase CHK1	4FT5	2.4	25, 27.4	-	27
H3K	Serine/threonine-protein kinase CHK1	4FT7	2.2	1	-	1
H4K	Serine/threonine-protein kinase CHK1	4FT9	2.2	20, 20.6	-	21
H5K	Serine/threonine-protein kinase CHK1	4FTA	2.4	40.4, 126	-	40
H6K	Serine/threonine-protein kinase CHK1	4FTC	2.0	23.2	-	-
ZAT	Serine/threonine-protein kinase CHK2	2W0J	2.05	-	-	200, 240
HCW	Serine/threonine-protein kinase CHK2	2YCR	2.2	-	-	69.6
3GG	Stromal cell-derived factor 1	4UAI	1.9	-	-	-
6V3	TAK1 kinase—TAB1 chimera fusion protein	5GJD	2.79	-	12	-
9DP	Tyrosine-protein kinase ABL1	3QRK	2.3	-	-	57
VSA	Tyrosine-protein kinase HCK	3VA1	2.46	-	-	-
1U2	Urokinase-type plasminogen activator	4FUH	1.6	610, 631	-	-
LIF	Vascular endothelial growth factor receptor 2	1YWN	1.71	-	-	3, 15.49
GIG	Vascular endothelial growth factor receptor 2	2OH4	2.05	-	-	3.5
42Q	Vascular endothelial growth factor receptor 2	3VHE	1.55	0.02, 23	19	0.03, 46
BAX	Vascular endothelial growth factor receptor 2	3WZE	1.9	0.02, 22	33, 93	0.16, 1210

### Appendix A.1. Motifs of Nonbonded Interactions

As shown in Figure A1, hydrogen bonding motifs originating from the diaryl urea moiety (Motifs 1–16) involve the N and O atoms of the urea group, which act as hydrogen bond donor and acceptor, respectively. In many cases, dual hydrogen bonding occurs. Motifs 17–20 represent hydrogen bonds originating from the R groups. Salt bridge motifs (Motifs 21 and 22) involve the interaction of positively charged basic residues (Lys) or negatively charged acidic residues (Asp) with oppositely charged groups of the DUs, originating from the R groups in both cases.

Motifs of cation–π interactions feature aromatic rings of DUs interacting with the positively charged side chains of Lys and Arg. In Motifs 23–28, the aromatic groups originate from the diaryl urea moiety, whereas in Motifs 29–34, they are derived from the R groups. π–π stacking interactions occur between the aromatic groups of the diaryl urea moiety (Motifs 35–51) or the R groups (Motifs 52–58) and the aromatic residues Phe, Tyr, and Trp in protein binding pockets. A wide spectrum of π–π stacking angles are sampled, ranging from parallel displaced to T-shaped configuration, and everything in between.

CH–π interactions involve the aromatic rings of DUs interacting with CH groups of aliphatic residues such as Ala, Leu, and Ile. The aromatic rings originate from the diaryl urea moiety in Motifs 59–84 and from the R groups in Motifs 85–95. XH–π interactions are represented by OH–π (Motifs 96–98), SH–π (Motifs 99 and 100), and NH–π (Motifs 101 and 102) interactions.
