# Supplementary material for: Molecular Recognition of Diaryl Ureas in Their Targeted Proteins—A Data Mining and Quantum Chemical Study
Source: Molecules. 2025 Feb 21;30(5):1007. doi: 10.3390/molecules30051007 (PMC11902014; doi:10.3390/molecules30051007)
Supplement: Supplementary file 1 [file molecules-30-01007-s001.zip › molecules-3465614-supplementary.pdf]

Figure S1. Structures of the 150 Diaryl ureas inhibitors analyzed in this thesis.

055 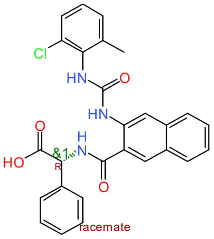	0BU 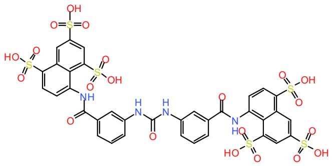	0F4 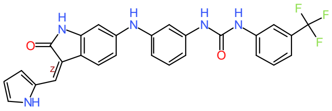
1K9 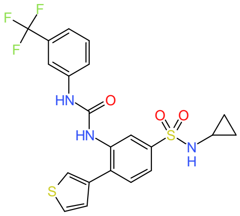	1PU 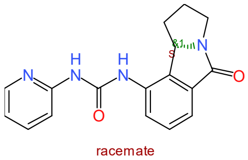	1U2 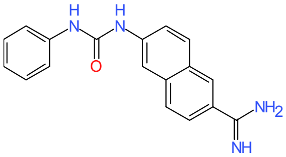
245 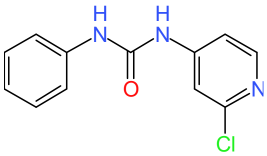	25D 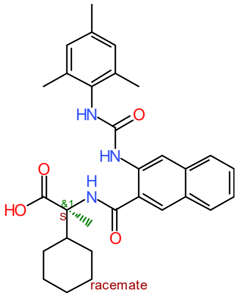	26B 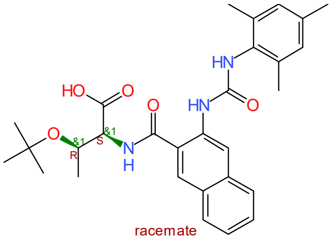

(Continued)

Figure S1:*(Continued)*

2OW 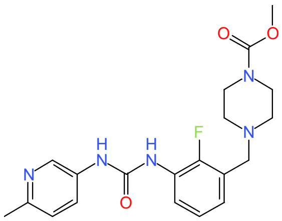	2X1 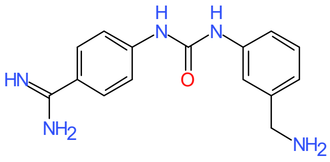	351 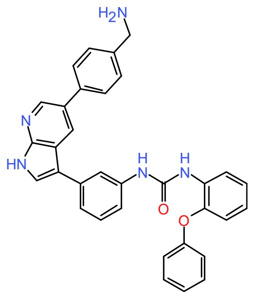
3GG 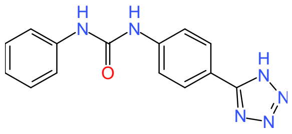	3WR 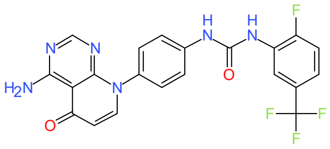	42Q 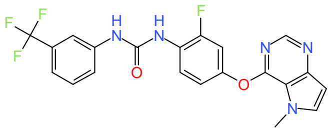
5KY 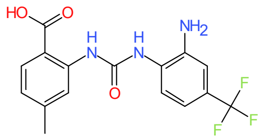	5LP 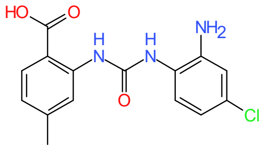	5O0 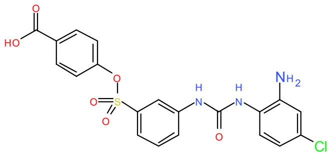
5O3 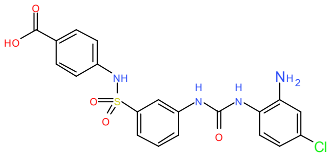	68Q 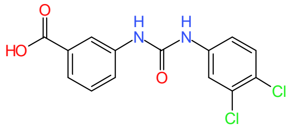	68V 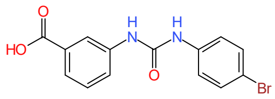

Figure S1:*(Continued)*

68W 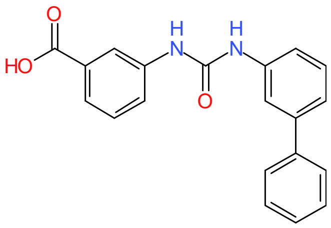	6EC 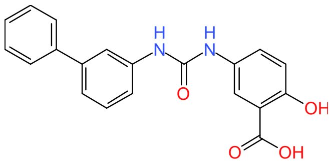	6EQ 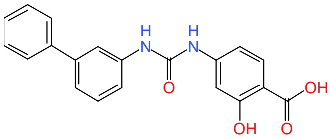
6G3 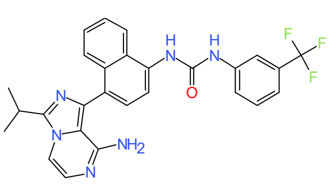	6K0 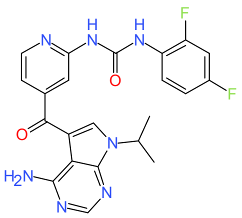	6LU 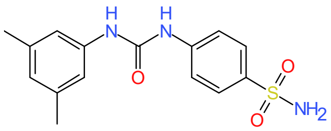
6UJ 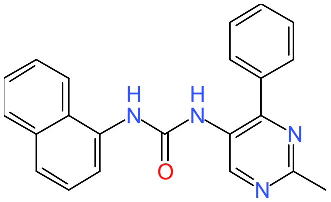	6UM 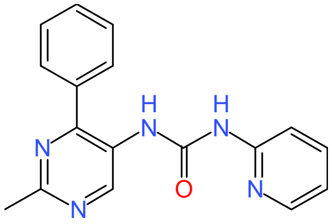	6UX 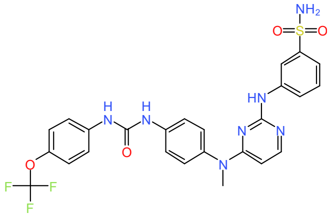
6V3 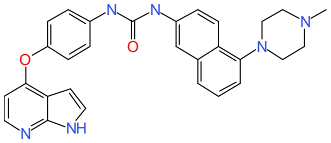	6X1 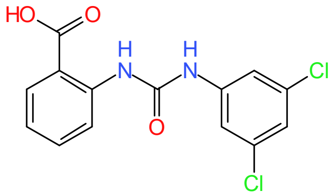	77A 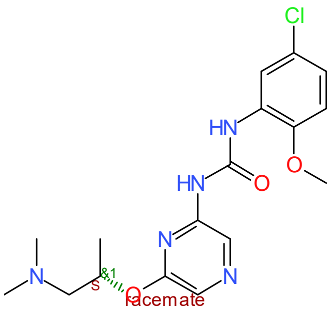

(Continued)

Figure S1:*(Continued)*

85A 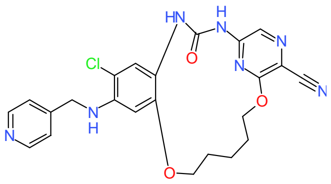	A25 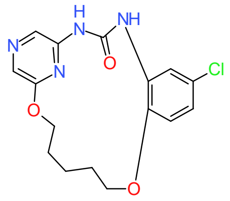	AKI 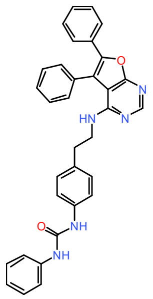
AU6 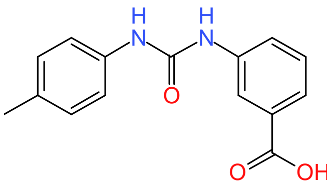	AYX 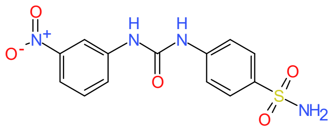	B10 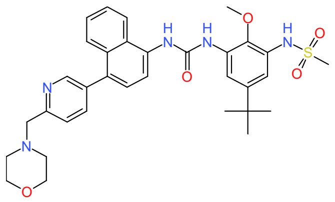
B6E 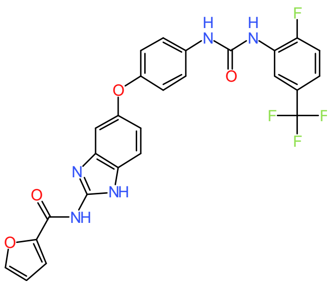	BAX 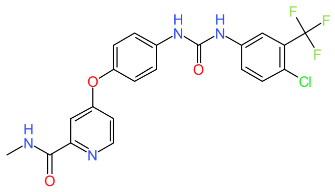	BUR 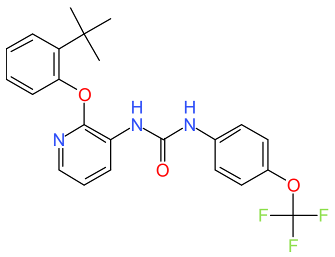
BW8 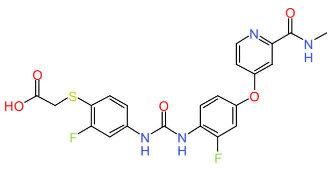	C4V 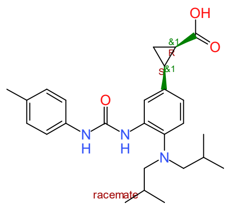	CJ5 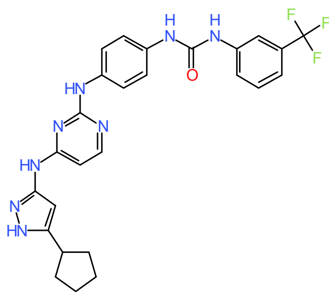

Figure S1:*(Continued)*

CW5 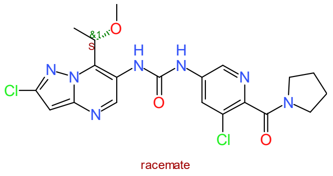	D3B 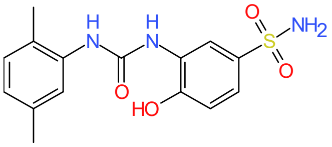	EON 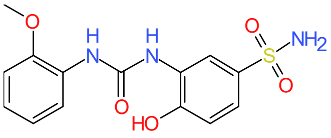
GIG 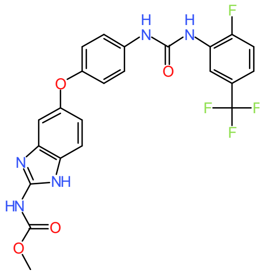	GP6 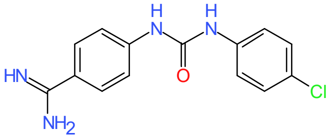	GP8 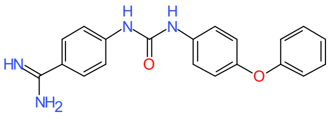
GW8 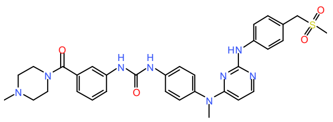	H1K 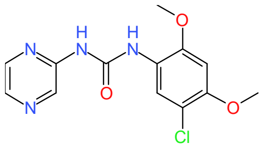	H2K 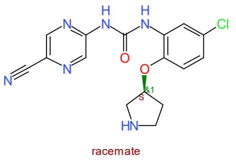
H3K 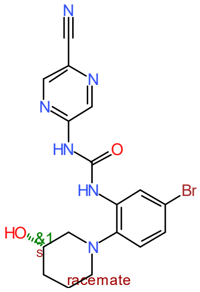	H4K 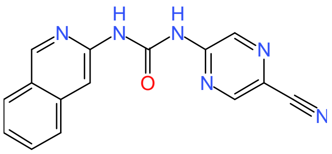	H5K 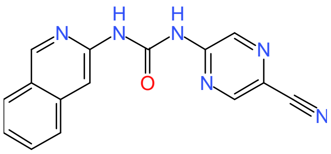

Figure S1:*(Continued)*

HCW 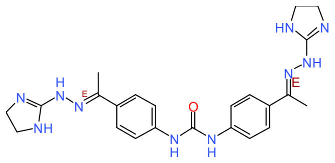	HQM 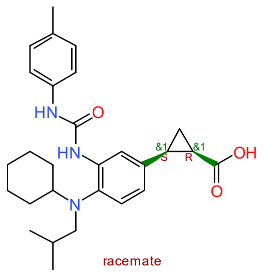	J3V 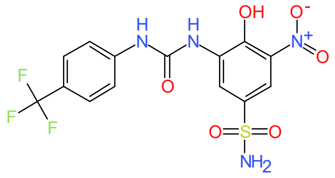
J4D 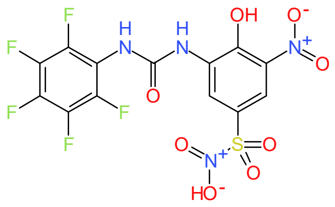	J6V 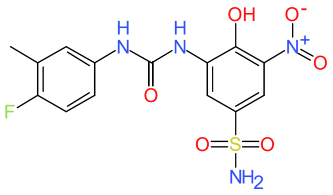	J7G 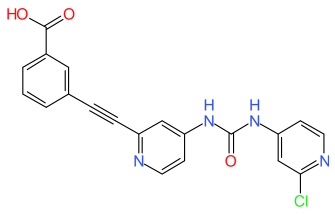
JHJ 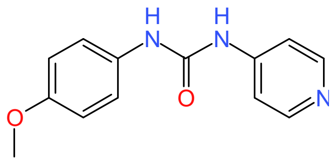	L37 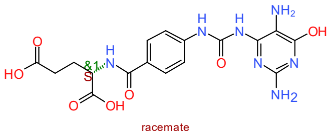	L64 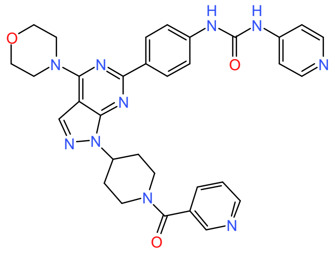
LIF 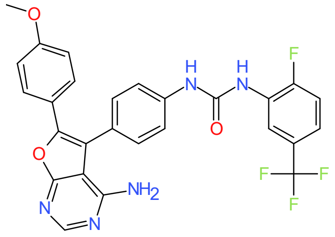	LQ5 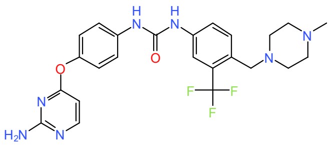	NSC 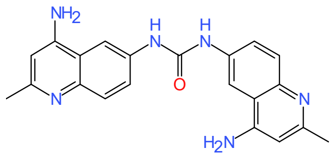

(Continued)

Figure S1:*(Continued)*

P9B 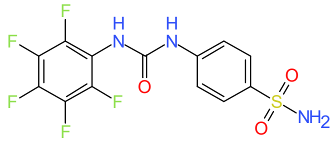	PD3 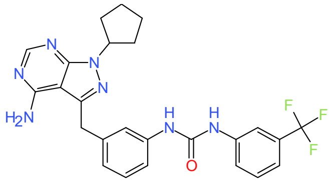	PD5 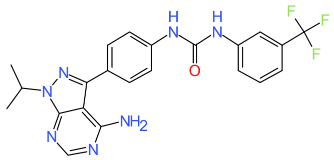
Q3B 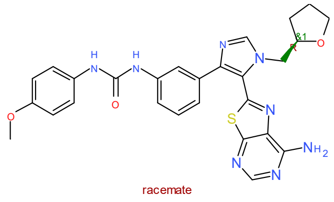	Q7M 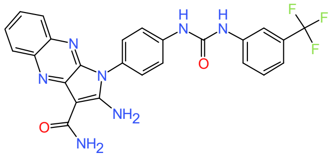	RC4 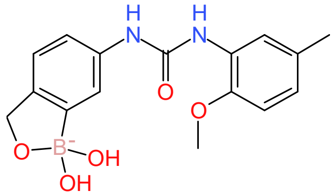
S16 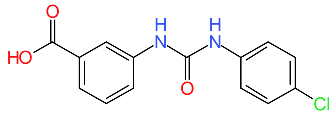	SVR 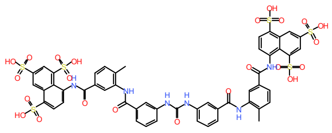	T6E 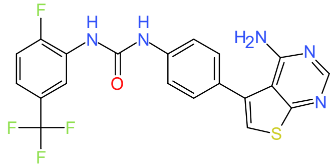
VSA 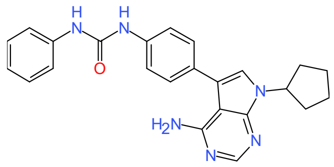	WPH 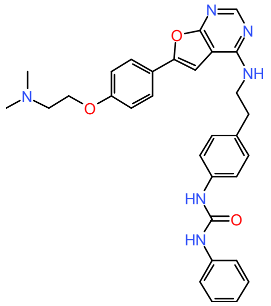	WWV 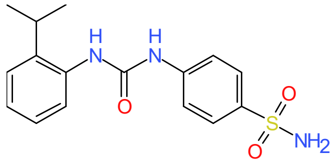

(Continued)

Figure S1: *(Continued)*

WWZ 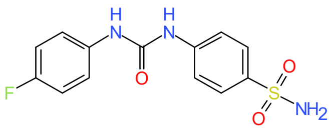	YPH 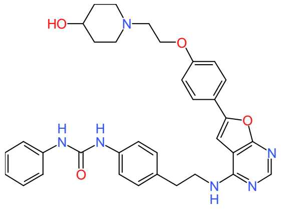	ZAT 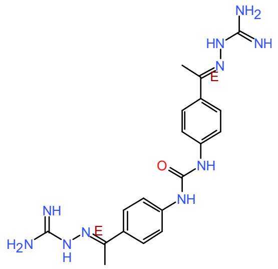
0YJ 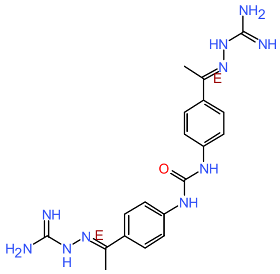	1AU 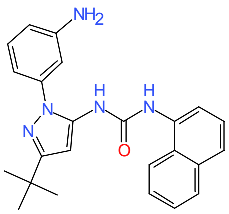	1AW 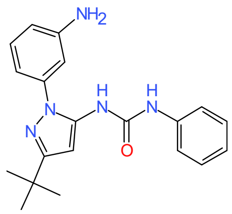
1BU 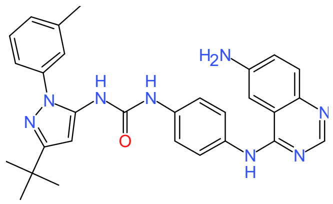	1PP 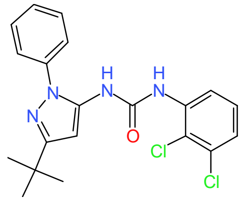	2PU 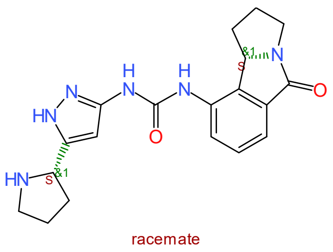
34X 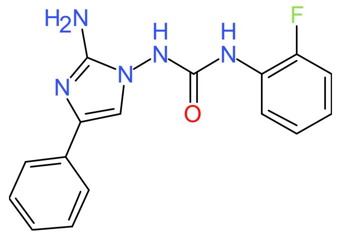	36K 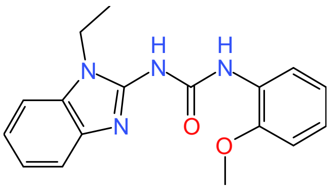	3H8 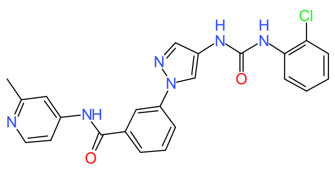

(Continued)

Figure S1:*(Continued)*

3HJ 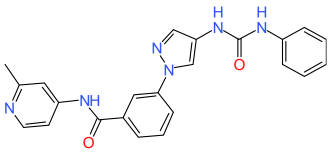	3HN 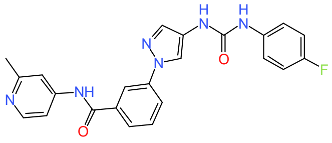	3HQ 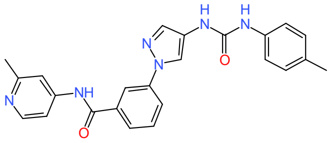
3NL 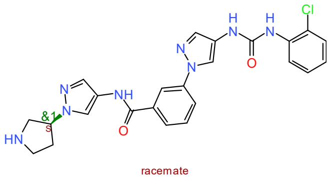	3O6 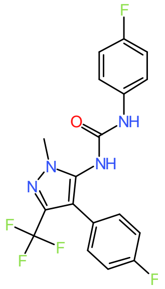	437 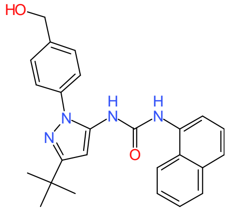
4R5 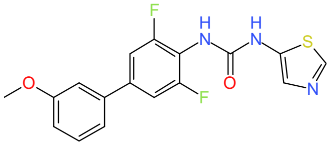	729 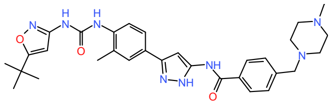	919 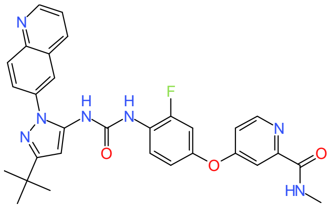
9DP 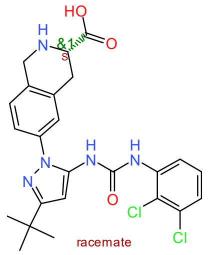	AK1 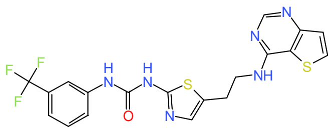	AK2 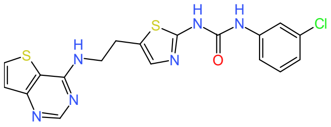

(Continued)

Figure S1:*(Continued)*

AK3 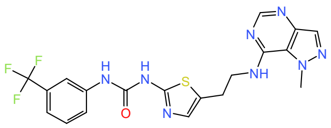	AK4 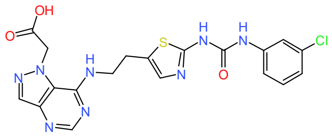	AK7 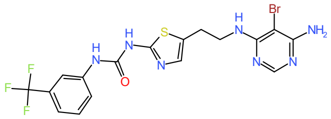
AQM 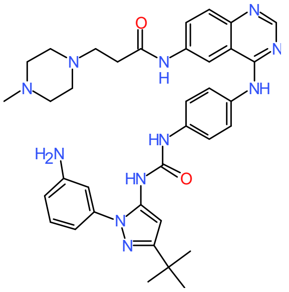	B96 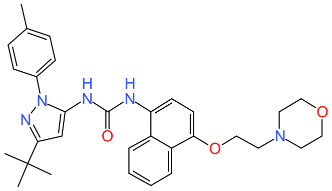	BMU 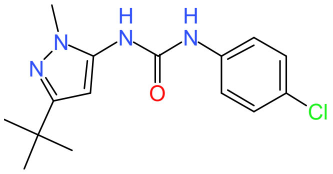
EDZ 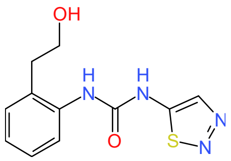	FDZ 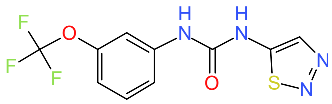	FXE 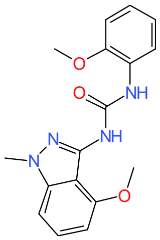
G2G 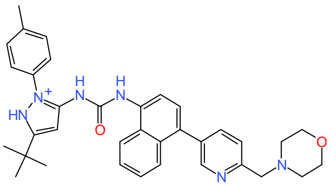	H6K 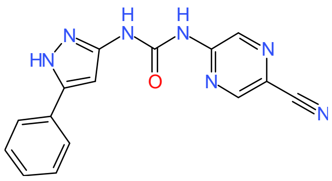	HIZ 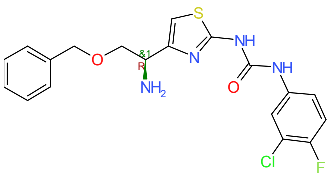 racemate

(Continued)

Figure S1:*(Continued)*

J7J<br>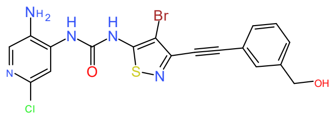	JK1<br>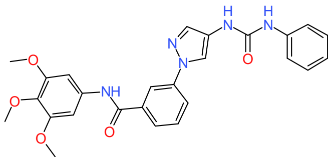	K9Y<br>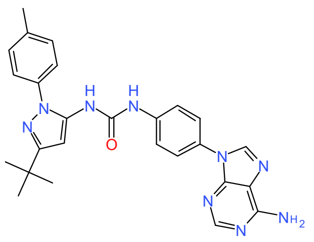
KAO<br>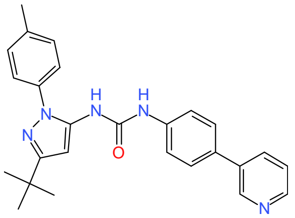	L09<br>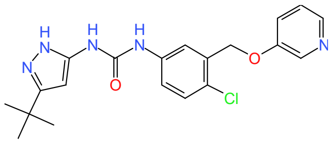	L0E<br>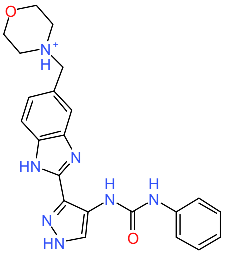
L51<br>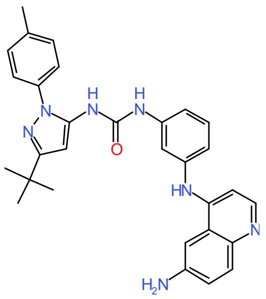	N58<br>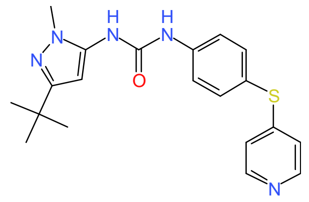	N61<br>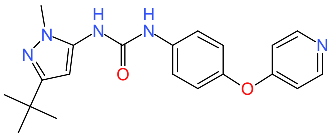
P5K<br>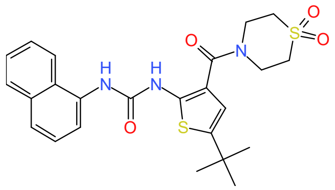	P78<br>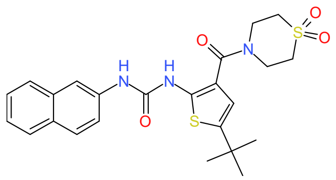	P79<br>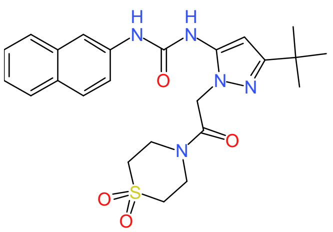

(Continued)

Figure S1:*(Continued)*

P7A 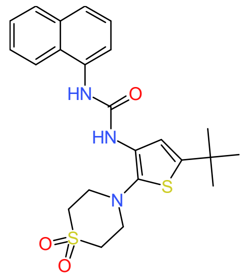	P7B 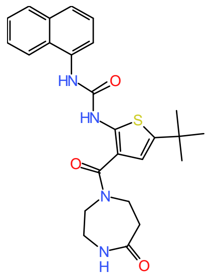	P7C 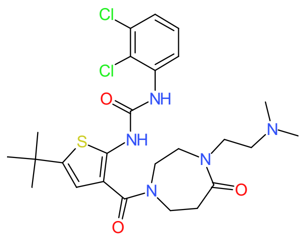
Q1A 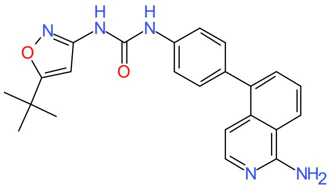	Q7U 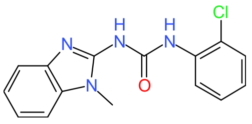	R24 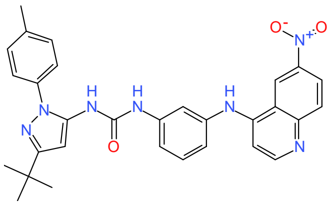
R39 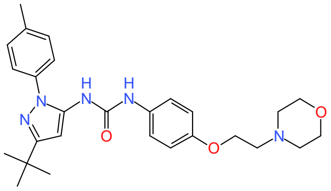	R48 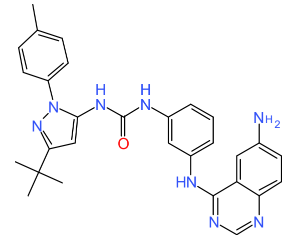	R49 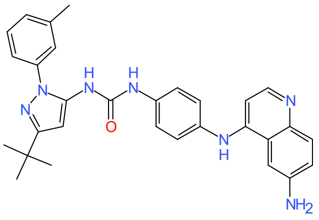
SR8 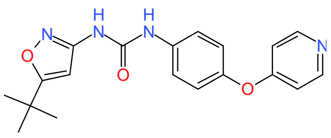	SS6 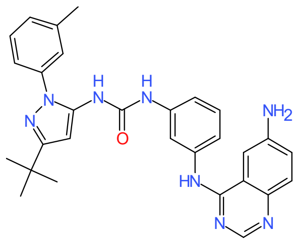	0YH 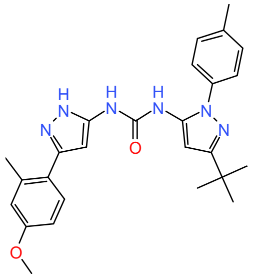

(Continued)

Figure S1:*(Continued)*

DG7 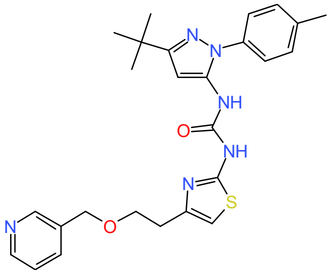	Z83 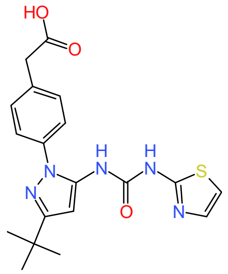	Z84 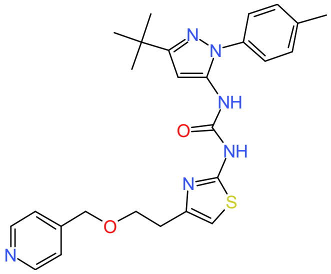
Z86 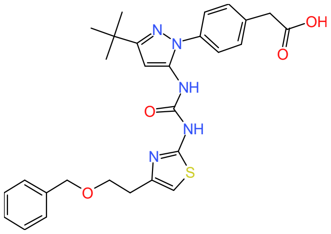	Z87 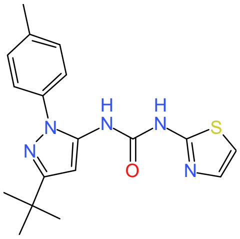	AK8 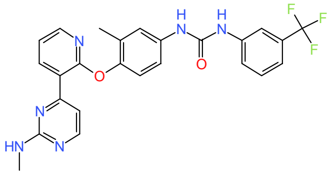
0F6 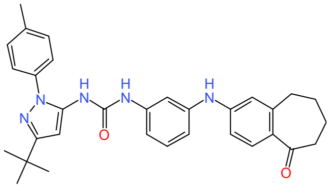	34Y 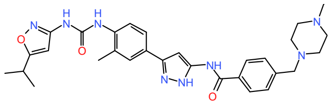	KIN 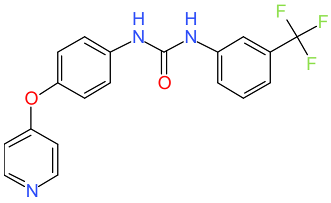

Table S1 List of representative interacting pairs in diaryl ureas bound protein complexes.

No.	Interaction	PDB ID	Angle	Distance	Intermolecular pair[a]
1	H-bonding	1DIG	141.43	3.30	L137 3001(A)…K56(A)
2	(Diaryl urea)	4O95	157.44 145.59	2.72 3.19	245 602(A)…D170(A)
3		6E43	150.75 130.40	3.04 3.20	HQM 501(A)...S167(A)
4		5HMR	155.59 142.55	2.70 3.16	FDZ 602(A)…D170(A)
5		3O8T	161.35 131.92 163.22	2.75 3.37 3.04	BMU 361(A)…E71(A)
6		1GII	162.38 167.93	2.83 2.85	1PU 501(O, N) (A)…V83(A)
7		2E9U	172.67	2.98	A25 1001(A)…E85(A)
8		2E9U	175.85	2.90	A25 1001(A)…C87(A)
9		4PA0	149.14	3.13	2OW 1101(A)…A91(A)
10		3P7C	170.39	2.87	P7C 362(A)…D168(A)
11		3D14	162.75	3.11	AK1 1(A)…K175(A)
12		4WI1	164.42	2.71	3O6 801(A)…Y266(A)
13		6EIM	151.66 149.34	2.81, 2.89	B6E 405(A)…E81(A)
14		4P5Z	158.28, 136.84	2.90, 3.12	Q7M 1001(A)…E670(A)
15		3D14	177.05 169.86 129.70	2.78 2.92 3.49	AK1 1(A)…E194(A)
16		5ALI	163.45	2.72	Q3B 1553(A)…Y466(A)
17	H-bonding	4FT7	178.37	3.06	H3K 301(A)…K38(A)
18	(R groups)	5I6D	171.48	2.90	AU6 402(A)…Q151(A)
19		5I6D	158.51	3.09	AU6 402(A)…S79(A)
20		6EEA	159.19 158.73	2.72 2.86	J3V302(O, N) (A)…T199(A)
21	Salt bridge	1BJU	-	2.86	GP6 910(A)…D189(A)
22		5EC8	-	2.80	5LP 302(A)…R84(A)
23	Cation-π	4PA0	-	3.02	2OW 1101(A)…R712(A)
24	(Diaryl urea)	5I6D	-	3.39	AU6 402(A)…K51(A)
25		3LFF	-	4.88	Z83 362(A)…K53(A)
26		4J8M	-	3.59	CJ5 501(A) K162(A)

(Continued)

Table S1 *(Continued)*

No.	Interaction	PDB ID	Angle	Distance	Intermolecular pair
27	Cation-π	4UAI	-	3.29	3GG 101(A)…R47(A)
28	(Diaryl urea)	4AOT	-	4.30	GW8 1319…K65(A)
29	Cation-π	2BAJ	-	3.78	1PP 401(A)…R67(A)
30	(R groups)	2W1E	-	3.74	L0E 1390(A)…R137(A)
31		3GCV	-	5.15	SS6 361(A)…R70(A)
32		3LFF	-	4.66	Z83 362(A)…K53(A)
33		3LFF	-	3.24	Z83 362(A)…R70(A)
34		4JBO	-	3.19	WPH 501(A)…R137(A)
35	π-π	1DIG	9.34	3.95	L37 3001(A)…Y52(A)
36	(Diaryl urea)	2E9U	171.62	4.51	A25 1001(A)…Y86(A)
37		4O95	62.06	3.60	245 602(A)…W386
38		4PYQ	66.1	3.80	2X1 301(A)…W186(A)
39		5I7A	143.23	3.99	68Q 401(A)…Y152(A)
40		5HMR	99.26	3.59	FDZ 602(A)…Y487(A)
41		3V5Q	63.95	3.55	0F4 902(A)…F698(A)
42		4JBO	135.69	4.74	WPH 501(A)…W277(A)
43		4JBO	81.05	3.22	WPH 501(A)…F144(A)
44		4X3J	155.1	3.74	3WR 1201(A)…F983(A)
45		4XNV	95.39	3.58	BUR 1101(A)…F62(A)
46		5A14	129.66	3.31	LQ5 1297(A)…F80(A)
47		5JFS	18.22	3.43	6K0 4000(A)…F589(A)
48		5KMO	130.45	3.49	6UM 901(A)…F589(A)
49		5KMO	169.21	3.57	6UM 901(A)…Y591(A)
50		4WI1	78.84	4.49	306 801(A)…Y285(A)
51		3LFF	133.51	3.78	Z83 362(A)…F169(A)

(Continued)

Table S1 *(Continued)*

No.	Interaction	PDB ID	Angle	Distance	Intermolecular pair
52	π-π	3GCV	93.37	3.62	SS6 361(A)…F169(A)
53	(R groups)	4JBO	177.37	3.79	WPH 501(A)…Y212(A)
54		4AT4	51.62	3.47	T6E 1839(A)…F711(A)
55		4AT4	161.59	3.34	T6E 1839(A)…Y635(A)
56		3D14	173.29	4.23	AK1 1(A)…Y225(A)
57		4EYJ	19.4	4.33	N61 401(A)…F109(A)
58		3V5Q	6.11	3.65	0F4 902(A)…Y619(A)
59	CH-π	5LMD	-	3.82	RC4 302(A)…L198
60	(Diaryl urea)	2YCR	-	3.68	HCW 600(A)…L226(A)
61		5N69	-	3.39	2OW 904(A)…L770(A)
62		4FT7	-	3.89	H3K 301(A)…V23(A)
63		1GII	-	3.42	1PU 501(A)…I10(A)
64		3P7C	-	3.94	P7C 362(A)…L75(A)
65		3O8T	-	4.04	BMU 361(A)…L75(A)
66		5HMR	-	3.94	FDZ 602(A)…L452(A)
67		5HMR	-	4.95	FDZ 602(A)…A450(A)
68		4FT7	-	3.53	H3K 301(A)…L84(A)
69		1GII	-	3.41	1PU 501(A)…L134(A)
70		2E9V	-	3.48	85A 1001(A)…L137(A)
71		2OH4	-	4.09	GIG 303(A)…V914(A)
72		3EFW	-	4.11	AK8 404(A)…L178(A)
73		3V5Q	-	3.53	0F4 902(A)…L591(A)
74		3V5Q	-	3.80	0F4 902(A)…L592(A)
75		3VHE	-	4.52	42Q 1170(A)…V899(A)
76		3VHE	-	3.73	42Q 1170(A)…V899(A)
77		4AOT	-	4.55	GW8 1319…I67(A)

(Continued)

Table S1 *(Continued)*

No.	Interaction	PDB ID	Angle	Distance	Intermolecular pair
78	CH-π	4UAI	-	3.49	3GG 101(A)…L42 (A)
79	(Diaryl urea)	5JFS	-	3.71	6K0 4000(A)…L567(A)
80		5JFS	-	3.40	6K0 4000(A)…L564(A)
81		6EIM	-	3.64	B6E 405(A)…E81(A)
82		3HV6	-	4.13	R39 361(A)…L74(A)
83		4WI1	-	4.49	3O6 801(A)…L276 (A)
84		3LFF	-	3.74	Z83 362(A)…K53(A)
85	CH-π	3EFW	-	3.64	AK8 404(A)…L263(A)
86	(R groups)	1YWN	-	3.69	LIF 301(A)…A864(A)
87		4AOT	-	3.52	GW8 1319…I67(A)
88		4P5Z	-	3.66	Q7M 1001(A)…V635(A)
89		4X3J	-	3.48	3WR 1201(A)…L971(A)
90		5A14	-	4.17	LQ5 1297(A)…V18(A)
91		5JFS	-	3.55	6K0 4000(A)…L567(A)
92		5JFS	-	4.04	6K0 4000(A)…V524(A)
93		3GCS	-	3.69	BAX 401(A)…L74(A)
94		4W4W	-	3.57	3HJ 501(A)…I70(A)
95		6ES0	-	3.43	BW8 401(A)…A45(A)
96	OH-π	5I6D	-	3.12	AU6 402(A)…S79(A)
97		4PA0	-	5.12	2OW 1101(A)…T94(A)
98		5I6D	-	3.54	AU6 402(A)…Y152(A)
99	SH-π	4PA0	-	4.49	2OW 1101(A)…M92(A)
100		3KVK	-	4.05	6X1 401(A)…M43
101	NH-π	1DIG	-	4.73	L137 3001(A)…Q100(A)
102		5I6D	-	3.68	AU6 402(A)…Q151(A)

Note: The associated 3D motifs are displayed in Figure S1.

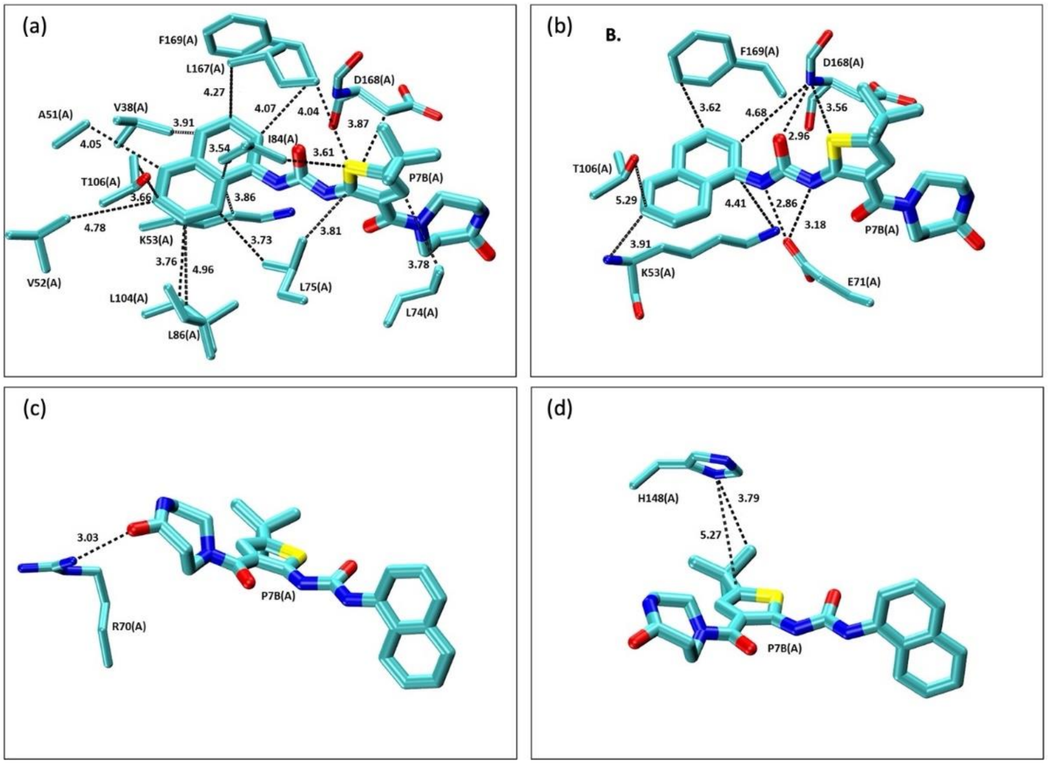

Figure S2. Three dimensional arrangement of P7B and its interacting residues of Mitogen-activated protein kinase 14 based on the 1.90 Å resolution X-ray crystal structure (PDB ID: 3P7B). (a) Residues involved in CH-π interactions with the diaryl urea moiety, (b) residues involved in other intermolecular interactions with the diaryl urea moiety, (c) interactions involving only R groups of the diaryl urea, and (d) residues interacting with both the diaryl urea moiety and R groups. Chain identifier for each residue is presented in parentheses.

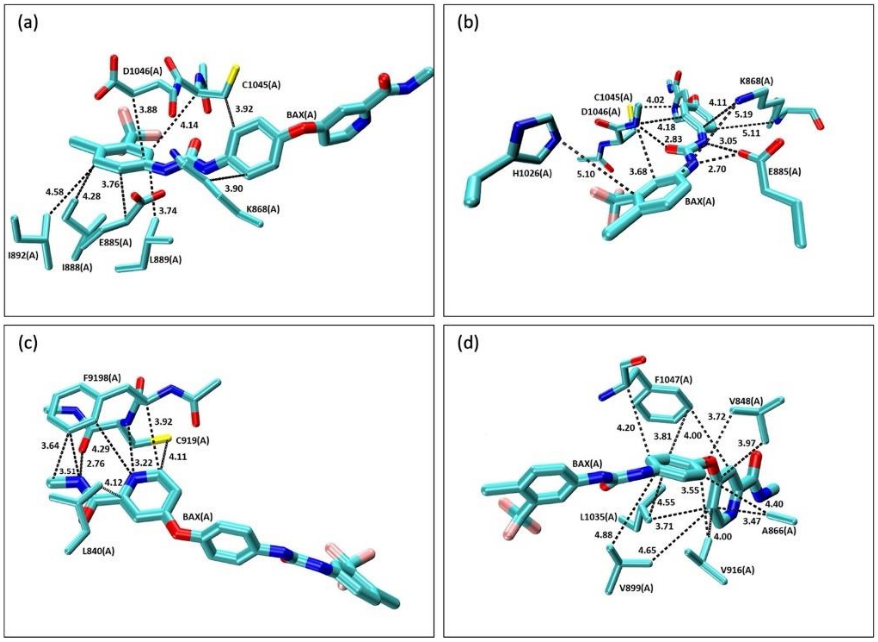

Figure S3. Three dimensional arrangement of BAX (sorafenib) and its interacting residues of vascular endothelial growth factor receptor 2 based on the 1.90 Å resolution X-ray crystal structure (PDB ID: 3WZE). (a) Residues involved in CH-π interactions with the diaryl urea moiety, (b) residues involved in other intermolecular interactions with the diaryl urea moiety, (c) interactions involving only R groups of the diaryl urea, and (d) residues interacting with both the diaryl urea moiety and R groups. Chain identifier for each residue is presented in parentheses.
